# Quantifying progress in research topics across nations

**DOI:** 10.1038/s41598-023-31452-8

**Published:** 2023-03-23

**Authors:** Kimitaka Asatani, Sumihiro Oki, Takuya Momma, Ichiro Sakata

**Affiliations:** 1 Department of Engineering, University of Tokyo, Tokyo, Japan; 2 Amsterdam School of Historical Studies, Universiteit van Amsterdam, Amsterdam, The Netherlands; 3 School of Humanities, Kwansei Gakuin University, Nishinomiya, Japan; 4 Japan Society for the Promotion of Science, Tokyo, Japan

**Keywords:** Computational science, Information technology

## Abstract

A scientist’s choice of research topic affects the impact of their work and future career. While the disparity between nations in scientific information, funding, and facilities has decreased, scientists on the cutting edge of their fields are not evenly distributed across nations. Here, we quantify relative progress in research topics of a nation from the time-series comparison of reference lists from papers, using 71 million published papers from Scopus. We discover a steady leading-following relationship in research topics between Western nations or Asian city-states and others. Furthermore, we find that a nation’s share of information-rich scientists in co-authorship networks correlates highly with that nation’s progress in research topics. These results indicate that scientists’ relationships continue to dominate scientific evolution in the age of open access to information and explain the failure or success of nations’ investments in science.

## Introduction

Bibliographic databases^1^, open journals^2^, and online educational content^3^ have liberated scientists from constraints on access to information. However, certain scientists or groups in hotspots of knowledge^4^ tend to produce more significant output^5^, while others follow their lead^6^. Pursuing trends is not the aim of science, and several studies have found that the development of non-conventional research is essential for generating new knowledge^7,8^. However, collective attention^9^ promotes community discussion and discovery^10^, and research that follows the trend is likely to have greater impact^11^. Recent developments in computational methods are helping scientists and funding agencies discover cutting-edge topics^12^ or assess the novelty of paper^13^.

Global investment in science^14^ has been narrowing the gap between nations, not only in terms of the number of published articles but also in the number of highly cited articles^15^. China has made significant strides in scientific research in recent decades^14^. The performance of a nation or region is assessed based on the structure of its research system, which is inferred from the output of each research field^16^. A recent study^17^ demonstrates that disparities in regional scientific competitiveness are being reduced through the analysis of the concentration of research fields. Conversely, the winners of major scientific awards^18^, top-performing research universities^14^, and high-impact publications^19^ remain confined to certain nations, such as the US and the UK. Several domain-specific studies^20–22^ have provided insight into the significant role played by certain nations in the development of domains. However, these microscopic analysis requires extensive effort and has not yet been generalized across all fields. Given that certain nations lead in science, several causes of national differences in scientific output have been analyzed: education systems^23^, social diversity^24^, and individual mobility^25^. As funding agencies are dedicated to selecting research topics^26^, it is essential to reveal the structural relationships and inequality between nations in terms of research topic.

In this study, we quantify national research topic progress using time-series comparisons of the references in published papers. The comparison identifies the microscopic difference between the research topics of nations. We assume that the aggregation of reference lists in papers from a nation represents its overall profile of engagement with research topics, as the reference lists are used for unsupervised^27^ and supervised^28^ estimations of research topics. Using 71 million research papers from Scopus, we identified a leading-following relationship among research topics between pairs of nations. For instance, China and Japan tend to engage in research topics that are similar to those in which the US and the UK previously engaged. Moreover, the accumulation of two-nation comparisons, which we define as the Topic Progress Index (TPI), reveals a long-term leading-following relationship between Western nations and Asian city-states, on the one hand, and other nations.

We also demonstrate that information-rich scientists (those with high eigenvector centrality in co-authorship networks) play a crucial role in steering the progress in research topics. From a co-authorship network of 16 million scientists, we identified information-rich scientists who are engaging in newer research topics that others follow, and who are likely to be cited more frequently. These information-rich scientists are often based in Western nations, and the proportion of information-rich scientists in many nations was correlated with research topics progress. These results provide support for national research strategies that promote global co-authorship^29^, the recruitment of top scientists^30^, and the encouragement of scientists to go abroad and return^31^.

## Results

### Research topic comparison between pairs of nations

Assuming the reference list of a paper indicates the research topic of the paper, (unregularized) vector representation of the research topics in nation A in year y can be introduced as $\textbf{T}^{\prime }_{\textbf{A},\textbf{y}}=(T_{1,A,y}^\prime ,T_{2,A,y}^\prime ,\ldots )$, where each element denotes the aggregation of references$1,\ 2\ldots$’s in nation A in year y. Each paper is assigned to its first author’s nation. We used tfidf weighting^32^ for aggregation to adjust the paper’s difference with respect to the number of references and to eliminate the effects of frequently cited references (detailed in “Methods” section). Then, we performed L2-normalization of $\textbf{T}^\prime _{\textbf{A},\textbf{y}}$ to obtain research topic $\textbf{T}_{A,y}$.

**Figure 1:**
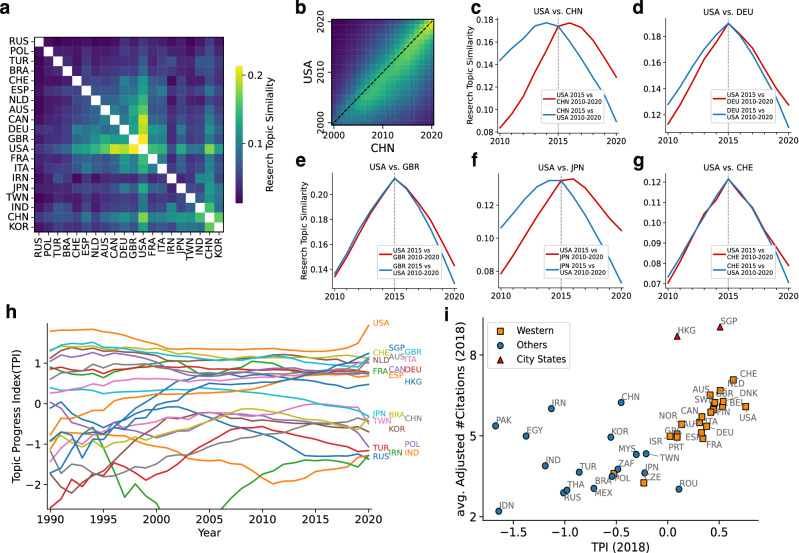
(**a**) Cosine similarity of research topic $\textbf{T}$ in 2015 between the top 20 paper-publishing nations. The order of the nations is determined by average linkage clustering. (**b**) The cosine similarity matrix of $\textbf{T}$ between China and the US from 2000 to 2020. (**c**–**g**) Two-nation comparisons: red lines indicate cosine similarities between $\textbf{T}$ in 2015 in the US and $\textbf{T}$ in 2010-2020 in China (**c**), Germany (**d**), the UK (**e**), Japan (**f**), and Switzerland (**g**). Blue lines indicate the opposite comparisons (in the other nation in 2015 and in the US in 2010-2020). (**h**) Yearly change in the TPI of the top 20 paper-publishing nations, plus Hong Kong and Singapore, from 1990 to 2020. (**i**) The average domain-adjusted citation count versus the TPI, both in 2018 for the top 40 paper-publishing nations. The figures were generated using matplotlib(3.6.0) and labeled with Illustrator(26.0.2).

In a comparison of research topic $\textbf{T}$ in 2015 between the top 40 paper-publishing nations, some groups of nations have a high similarity in research topics (Fig. 1a). The Anglophone nations (USA, GBR, CAN, DEU, etc.) tend to have a similar research topic, while the Asian nations (CHN, IND, KOR, etc.) have a weaker link. This suggests that the former nations may form the core group in research topics. However, it is unclear which group is leading in research topic. A time-series comparison of $\textbf{T}$ between nations reveals a time lag in research topic between them (China and the US comparison is shown in Fig. 1b). The research topic $\textbf{T}$ in China after 2015 is similar to the $\textbf{T}$ in the US in 2015 (red line in Fig. 1c), and $\textbf{T}$ in the US before 2015 is similar to $\textbf{T}$ in China in 2015 (blue line in Fig. 1c). Assuming that research topics neither undergo rapid change nor evolve in a looping process, the difference in slope between the two lines in Fig. 1c indicates the delayed adoption of research topics in China compared to the US. Japan also lagged behind the United States, whereas Germany was only slightly behind, and the United Kingdom and Switzerland showed no delay (Fig. 1d-g; other comparisons among the top seven nations are shown in Fig. S1). We note that the results that papers are assigned to nations by fractional counting^33^ (Fig. S2) show similar results that papers are assigned to the first author’s nations (Fig. 1).

### Quantifying research topic progress of nations

Cosine similarity (cos) is a metric of the similarity between two vectors of an inner product space and is the cosine of the angle between them. In this study, the leading-following relationship between two nations is derived from the time series comparison of the cosine similarity between $\textbf{T}$ of them (detailed in “Methods” section). As with the analysis of the US(A)-China(B) case (Fig. 1c), the difference between $cos(\textbf{T}_{{A},{y}},\textbf{T}_{{B},{y}^+})-cos(\textbf{T}_{{A},{y}},\textbf{T}_{{B},{y}^-})$ (change in red line) and $cos(\textbf{T}_{{B},{y}},\textbf{T}_{{A},{y}^+})-cos(\textbf{T}_{{B},{y}},\textbf{T}_{{A},{y}^-})$ (change in blue line) indicates the US’s progress in research topics. The TPI of a nation is an aggregation of the comparisons with all other nations weighted by their respective numbers of published papers, for time intervals $\Delta =1,\ 2,\cdots ,\ \tau$ years. We calculated TPI using $\textbf{T}$ of the top 40 paper-publishing nations during 2010-2020, with parameter $\tau =5$ years considering both rapidly changing domains such as computer science and others. Because the data were up to 2020, we calculated TPI around 2020 by masking the information after 2020 (detailed in “Methods” section).

The Western nations^34^ (Western Europe and English-speaking developed nations) and Asian city-states (Singapore and Hong Kong) had high TPI for decades relative to other nations (Fig. 1h), whereas the dispersion in the number of published papers of those nations settles over time (Fig. S3). Taiwan, South Korea, and Japan had low TPI values, but their research topics did not differ markedly from those of the US and UK (Fig. 1a). Conversely, Switzerland had high TPI values, but its research topics were not similar to those of the US or the UK (Fig. 1a), indicating that a convergence of research topics with the US was not a necessary condition for research topic progress.

TPI relates to the average domain-adjusted citations, except for some nations (Fig. 1i). The impact is adjusted by the average number of citations per domain (see the “Methods” section). While the average domain-adjusted citations for China and the United States are similar, TPI identifies a leading-following relationship between them. The high citation numbers for papers from Hong Kong and Singapore are ascribed to those nations’ highly selective practices for recruiting scientists. Relative levels of progress or delay in topic uptake among nations are observed in each nation’s evolution of citing high-impact papers (Fig. S4): the US and UK tend to cite such papers earlier than China and Japan do. The same trend is observed over the average time (within five years after publication) each nation took to cite the 1000 most-cited papers (Fig. S5). However, this naive indicator is biased toward the most-cited articles, and it does not quantify research topic progress until several years later.

**Figure 2:**
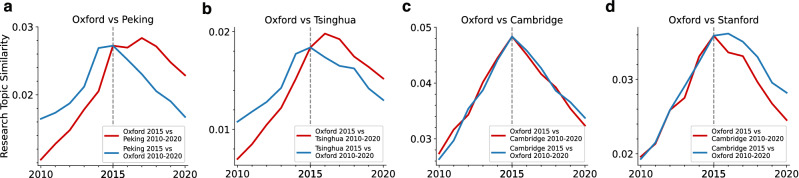
Detailed analysis of topic progress in universities: (**a**–**d**) University-level research topic comparison between Oxford and Peking University (**a**), Tsinghua University (**b**), Cambridge (**c**), or Stanford (**d**). The red lines indicate cosine similarities between $\textbf{T}$ in 2015 in the Oxford and $\textbf{T}$ in 2010-2020 in other universities. Blue lines indicate the opposite comparisons.

Next, we compare the university’s research topic progress to that of Oxford, which is ranked as the top university in the world^35^. Peking University and Tsinghua University lagged behind Oxford University (Fig. 2a,b). However, the University of Cambridge (Fig. 2c) did not, and Stanford University(Fig. 2d) slightly progressed to Oxford University. Other results shown in Fig. S6 indicate that top universities’ progress in research topics aligns with those of their nation. This result indicates that the topic progress of each nation might not correspond with the percentage of high-level universities within it.

**Figure 3:**
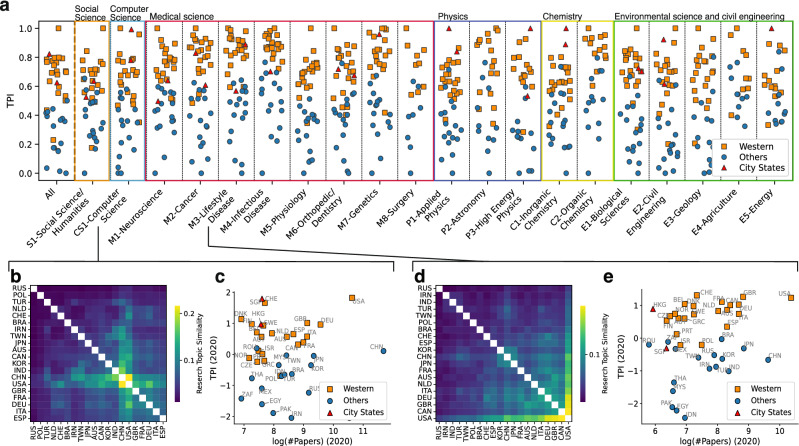
Detailed analysis topic progress in each domain. (**a** Strip )plot of TPI (normalized 0 to 1) of nations in 2020 (in 2019 for M4-Infectious Diseases) in whole domains and in each of the 20 domains. Nations that published less than 300 papers during the year in each domain are excluded. (**b**) Cosine similarity of $\textbf{T}$ between the top 20 nations in the number of papers in 2020 sorted by average linkage clustering in CS1-Computer Science. (**c**) Detailed plot of domains: the number of papers and TPI for each nation in CS1-Computer Science. (**d**, **e**) Same plots of (**b**, **c**) for M3-Lifestyle Disease. The figures were generated using matplotlib(3.6.0) and labeled with Illustrator(26.0.2).

The research topic progress of the Western world was observed in every domain (Fig. 3a; domain detail is shown in Supplemental Table T1). Note that some perturbed periods at specific domains are excluded (Supplemental Table T2). The research topics of Asian nations and Western nations differ in several domains, but they are similar in others (Supplemental Fig. S7). For example, Chinese/Indian research topics in the M3-Lifestyle Disease domain differ from those of the US and UK (Fig. 3d) and lag behind them (Fig. 3e). In contrast, in the CS1-Computer Science domain, China and India conduct research similar to the US and UK (Fig. 3b) but lag behind them (Fig. 3c). The similarity indicates that open access to information and the absence of geographical restrictions in the domain may synchronize the research topic, but the time lag remains.

### Information-rich scientists and research topic progress

Because of the slight differences in accessible information, the information spread among scientists may determine their research topic. Not surprisingly, the research topics of scientists resemble those of their co-authors (Fig. S8). Therefore, co-authorship networks entail a process of dissemination of research topics between scientists. We analyze a co-authorship network consisting of 16 million authors with 395 million relationships. Assuming that the amount of information value a scientist transmits via a link to another scientist is proportional to the amount of information value received, the extent of information value convergence to the node is calculated as the eigenvector centrality^36^. Centrality is used to estimate economic outcomes/social status^37,38^ and detection of the active part of the brain^39^. For comparison, we also calculated PageRank^40^, which gives more weight to a central node in small subgraphs; degree centrality; and the number of previously published papers.

**Figure 4:**
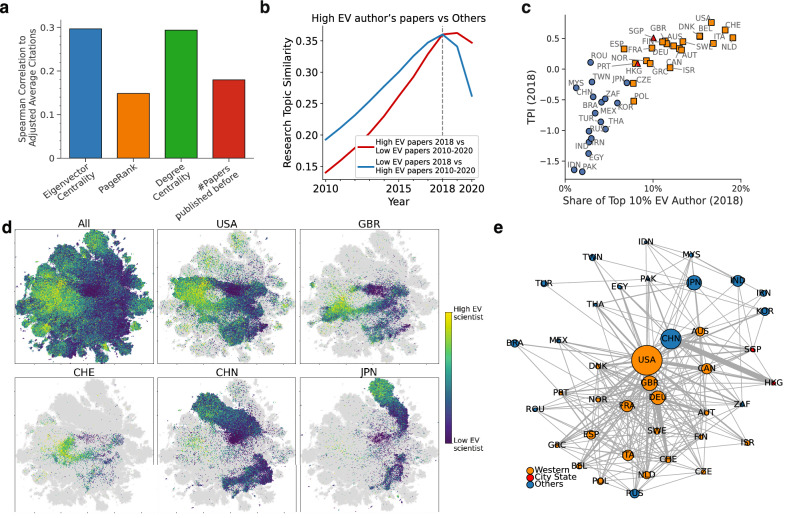
Information spreading on co-authorship network and research topic progress. (**a**) Blue, orange, green, and red bars show the Spearman correlation coefficients between the domain-adjusted citation count and eigenvector centrality, PageRank, degree centrality, and number of previously published papers for each author, respectively. (**b**) Comparison of $\textbf{T}$ for the top 50% of papers (on the basis of the highest author eigenvector (EV)) and bottom 50% papers. The comparison is based on the year 2018. (**c**) The relationship between the TPI (2018) and the percentage of authors with the top 10% eigenvector centrality (2018) for each nation. (**d**) Visualization of the co-authorship network: Each scientist is colored in accordance with eigenvector centrality (yellow indicates high, and blue indicates low). The 2D position is obtained by UMAP^41^ from the 128-dimensional LINE^28^ embedding of the network. The authors of all nations (top left) and of five selected nations are plotted. (**e**) The network of nations based on international co-authorship density. Each edge is weighted by the number of co-authorship links between the pair of nations divided by the lower number of authors among the two nations. Node size indicates the number of authors in each nation. The figures were generated using matplotlib(3.6.0) and labeled with Illustrator(26.0.2).

The eigenvector centrality and degree on the 1999-2018 co-authorship network are correlated with the average domain-adjusted citations (Spearman R $=$ 0.297 and 0.294, respectively; Fig. 4a). The higher citation performance of high-degree scientists indicates that a large team or many collaborations increases scientific impact. However, the correlation of PageRank with citation impact is lower. This indicates that the local central position (lab leader, group leader, etc.) within a small sub-network (team or small community) is not critical to citation performance. Eigenvector centrality is not much affected by the scientist’s position in a small sub-network but rather by the information convergence in the whole network. Therefore, the correlation between impact and eigenvector centrality indicates the importance of connectivity to the core scientists of the entire co-authorship network. Moreover, research topics of papers written by high-eigenvector authors progress in research topic compared to those of other papers (Fig. 4b). However, the difference between centralities is not significant (Fig. S9).

After aggregation of scientists on a national scale, the only feature that correlates strongly with a nation’s research topic progress is the proportion of high eigenvector scientists. The proportion of authors with the top n% of eigenvector centrality values is strongly correlated (Spearman R $=$ 0.879, n = 10%) with the TPI in each nation (Fig. 4c), and the correlation is also high when n $=$ 1% or 20% (Fig. S10). However, authors with high values of degree centrality or PageRank display weaker correlations (Spearman’s R=0.787 ,n = 0.1% or r=0.568 ,n= 1%, respectively; Fig. S10). This result indicates that nations that have scientists located in a global information-spreading core advance in research topic.

The high-eigenvector-centrality scientists are illustrated by bright color in Fig. 4d. These scientists are located mainly in the middle left area. Scientists in the US, UK, and Switzerland are likely to be located in the same area (middle left of each figure in Fig. 4d), and the area is populated with a high percentage of high-eigenvector-centrality (yellow) scientists. By contrast, most Chinese and Japanese scientists plot separately in the peripheral areas. National differences in the proportion of high-eigenvector-centrality scientists are explained by the international co-authorship density (Fig. 4e). Western nation’s scientists frequently coauthor with scientists in other western nations. Other peripheral nations such as China and Japan have low collaboration with western nations, and collaboration in these peripheral nations is also rare. Therefore, scientific information is spread intensively among scientists in Western nations, and scientists in other nations are exposed to little valuable information.

## Discussion

The historical and global divide in research topic progress remains strong, despite the advancement of developing nations in science^42^ and the increased open access to scientific information^1–3^. Research topics originating in the Western World and city-state nations in Asia are later engaged with by the rest of the world, such as Japan, Brazil, and South Africa, consistent with many domain-specific analyses^20–22^. Interestingly, time-lags are observed in all the analyzed domains, including computer science, in which there are fewer geographical constraints on access to information and computing hardware.

The TPI correlates strongly with the percentage of information-rich scientists. This analysis explains why open nations (characterized by high co-authorship and mobility of scientists) have greater impact on science^2^. The UK and the US have highly ranked universities^43^ that educate top scientists who frequently conduct joint research with scientists at institutions in other nations^44^. These highly ranked UK and US universities attract notable international scientists^25^. To reduce the gap with the west, China encourages its scientists to conduct research abroad and then return to China^45^. At the end of the 1990s, Hong Kong and Singapore had successfully advanced research topics (Fig. 1h), demonstrating their cultivation of a productive research ecosystem^46^. Conversely, China and Japan were falling behind in research topics and had few information-rich scientists. This difference is consistent with the observation that a large, long-term investment in science does not necessarily result in a leading position in pioneering new research topics and trends. However, given the rapid expansion of the number of Chinese scientists and China’s government strategy^47^, future structural changes in co-authorship networks must be expected.

Analyses of culture, art, and business indicate that individual creativity is increased in networks or places where creative people congregate. For instance, a person obtains a higher income if at the center of a local community^48^, or that person becomes commercially more successful if he or she is close to the center of an art market^49^. Other analyses have demonstrated that the number of registered patents^50^ and talented parsons^51^ highlight the scale effects of collective creativity among regions or nations. A further study^52^ demonstrated a link between national performance and the centrality of its components (national products) in the estimated components-relationship network. Our study demonstrates the scale effect on creative outputs from a large-scale network of individual records of research activities.

A limitation of this study is that TPI cannot evaluate the topic progress of a group of scientists who have small numbers of publications. Because TPI assumes continuous changes in research topics, it is not valid for domains where the research topic is dynamically changing (such as in the study of infectious diseases in 2020). TPI is a quantification of the time-delay in science between some sets of papers, but it does not assure a causal relationship in the time-delay between them. To explain the emergence of delays in research topics across nations, a statistical model that generates a time-series of topic changes of nations needs to be developed.

TPI is not a direct indicator of each nation’s research creativity. Advanced research topics do not always generate creative outcomes, but the two factors are closely related in modern society. We need to analyze other factors that contribute research topic progress of nations. For example, TPI does not correlate with basic skills in reading, math, and science^53^, which indicates that students in nations whose research topics are delayed may lose their chance to conduct important research. Additionally, the high TPI in the Western World (Fig. 1h) might be facilitated by the ready availability of English-language skills in those nations. Paper’s language can influence citations^54^, and language skills may affect a scientist’s connection to central scientists who may speak English. These language barriers could be reflected in the structural divide of nations in the co-authorship network (Fig. 4d). It is also necessary to examine whether nationality bias plays a role in peer review, as has been demonstrated for gender bias^55^.

## Methods

To compare and quantify the research topics progress, we extracted the reference lists from all published papers indexed in Scopus. We estimate the topic of a paper from the tfidf value of the contained reference list. The aggregation of tfidf papers of year y at nation A is considered research topic $\textbf{T}_{A,y}$. Then, we conducted a time-series comparison of $\textbf{T}$ between nations and analyzed the progress/delay in research topics. Next, we simulated the information-spreading on a co-authorship network; in this part, with simple assumptions on information-spreading, we calculated the network centrality of the author.

**Figure 5:**
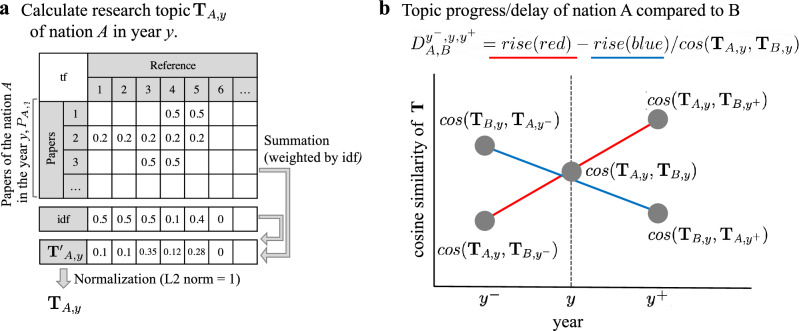
Calculation of Leading-Following Relationships Between Nations: (**a**) The research topic $\textbf{T}$ is based on references in the papers published in a particular year. We weight each reference by using the $\textrm{tfidf}$ framework. For each paper, $\mathrm {tf(r,p)}$ (the reference frequency of r in paper p) is the number of occurrences of r divided by the total number of references in the paper. We sum the values of $\textrm{tf}$ for $P_{A,y}$ (papers published in nation A during year y), weighted by the inverse document frequency $\textrm{idf}$ (as discussed in the “Methods” section). $\textrm{idf}$ indicates the rarity of the references (the amount of information the reference provides)^32^. (**b**) Topic progress/delay between pairs of nations: $D_{A,B}^{y^-,y,y^+}$ is calculated as the difference between the amount of rise of the red and blue lines. The red line indicates the extent to which nation B follows nation A (the blue line indicates the converse). In the example shown, research topics in nation A are followed by those in nation B.

### Data preprocessing

The Scopus dataset covers all domains of science. We use 70,731,510 papers from 1970 to 2020 categorized as articles, letters, reviews, and conference papers, excluding other forms of published documents, such as errata, conference reviews, and books. A few articles with no information on authors or affiliations were excluded. Note that authorship and affiliation are identified with high accuracy in Scopus^56^.

Internationally co-authored papers totaled 12,922,609. We adopted first author’s first affiliation protocol to select the nation where the main part of paper was conducted, as in most cases the contribution of the first author to a paper is significant. In the co-authorship analysis, we specified an author’s nation as the nation that appeared most frequently in the affiliations listed in the author’s publications in the preceding five years. If this protocol generated multiple nations for an author, the nation for the author was randomly assigned from among the multiple nations.

 We also perform fractional counting of papers^33^ for each nation to obtain robust results. (Fig. S2 shows the results using fractional counting). When using fractional counting, international co-authorship papers between two nations result in a high similarity in research topics comparison.

### Clustering and extraction of fields

The Scopus data include field labels, keywords, and journal categories of papers. The label of a published journal was used to estimate the field labels of papers published in that journal. However, multiple field labels and keywords were assigned to some papers. Furthermore, the recent development of interdisciplinary mega-journals made it difficult to categorize certain journals as belonging to one field.

Consequently, we adopted citation network clustering, because the reference list of the paper contains information about its domain. We used the Leiden method^57^ to cluster the papers on the citation networks consisting of 1,217,886,002 edges. We obtained 20 clusters (called domains) composed of more than 500,000 papers each, in the form of applied physics, infectious diseases, computer science, etc. We performed recursive clustering using the same method and obtained sub-clusters (sub-domains) for use in calculating the domain-adjusted citation count and in extracting key phrases. The details of the clusters and sub-clusters are presented in Supplemental Table T1.

### Calculating domain-adjusted citation count

The number of citations differs considerably across domains or sub-domains. For example, papers in the chemical and medical sciences tend to carry more citations than papers in the social sciences and humanities. To remove this inequality, the citation count of a paper was normalized by dividing it by the average citation count of the corresponding sub-domain. The mean of the domain-normalized citation count was then set equal to the mean of the original citation count for improving interpretability. Domain normalization is widely used as field-weighted citation impact^58^ and field-weighted citation impact^59^.

### tfidf Vector of References

References in a paper are associated with the research topic of that paper. Therefore, the aggregation of reference lists from papers published by a nation in a specific year represents the research topics in which the nation is engaged during that period. The multiset of references in a paper, operationalized as a bag of words (BOW) in natural language processing, is a straightforward representation of the research topic of the paper. However, there are a substantial number of highly cited references, and these highly cited references heavily influence the BOW of references. To avoid heavy influence of such references, we applied the $\textrm{tfidf}$ weighting framework to evaluate the amount of information that each reference(term) carried in a paper^32^.

Figure 5a shows the procedure to calculate research topic $\textbf{T}$. In Eq. (1), the value of $\textrm{tfidf}(r,p)$ is the product of the reference(term) frequency $\textrm{tf}(r,p)$, and the inverse paper(document) frequency.

1 $$\begin{aligned} \text {tf-idf}(r,p) = \text {tf}(r,p) \times \text {idf}(r,P_{all}) \end{aligned}$$

In Eq. (2), $e_{r,p}$ denotes the existence of reference r in paper p (if the reference is present, $e_{r,p}=1$, otherwise $e_{r,p}=0$). The quantity $\textrm{idf}(r,P_{all})$ indicates the rarity of the reference r in the entire set of papers $P_{all}$. In Eq. (3), $\textrm{idf}(r,P_{all})$ is the logarithmically scaled index of the maximum number of references appearing, $\max _{r^{\prime } \in P_{all}} n_{r^{\prime }}$ divided by $1+n_r$, where $n_r$ is the number of times that the reference *r* appears in $P_{all}$.

2 $$\begin{aligned} \text {tf}(r,p)= & {} \frac{e_{r, p}}{\sum _{r^{\prime } \in p} e_{r^{\prime }, p}} \end{aligned}$$

3 $$\begin{aligned} \text {idf}(r,P_{all})= & {} \log { \frac{\max _{r^{\prime } \in P_{all}} n_{r^{\prime }}}{1 + n_r}} \end{aligned}$$

In Eq. (4), $t_{r,A,y}^\prime$ (the prevalence of research including reference r for nation A in year y) is the sum of the $\textrm{tfidf}$ values for reference r over $P_{A,y}$ (all papers from nation A in year y). The list of research topics accommodating all references for nation A in year y is denoted as $\textbf{T}^\prime _{A,y}=(t_{1,A,y}^\prime ,t_{2,A,y}^\prime ,\ldots )$. $\textbf{T}^\prime _{A,y}$ was normalized so that its L2 norm was 1, and we obtained research topic $\textbf{T}_{A,y}=(t_{1,A,y},t_{2,A,y},\ldots )$ of nation A at year y.

4 $$\begin{aligned} {t^\prime }_{r,A,y} = \sum _{p \in P_{A,y}} \text {tf-idf}(r,p) \end{aligned}$$

Papers containing more than 100 or fewer than 5 references were omitted from the analysis to exclude review papers and incomplete data. We ignored references with citation numbers more than 1000 to prevent distortions of cosine similarity; these commonly cited references could not add meaningful information to the analysis because they were likely to be cited from a wide range of papers. This procedure is standard in calculating the $\textrm{tfidf}$ in natural language processing to enhance task performance^60^.

### Calculation of TPI

First, we considered the topic of influence between a pair of nations on a reference r at year y considering a rise from $y^-$ to $y^+$. Nation A’s degree of being followed by nation B on reference r is quantified as the product of $t_{r,B,y^+}-t_{r,B,y^-}$ (B’s increase of engagement on the topic from $y^-$ to $y^+$) and $t_{r,A,y}$ (A’s engagement with the topic at t). Consequently, the extent of A’s topic progress toward B with respect to reference r can be calculated from the difference between A’s degree of being followed by B and B’s degree of being followed by A [Eq. (5)]. When A or B does not engage with the research topics related to reference *r*, $d_{A,B}^{y^-,y,y^+}$ equals 0.

5 $$\begin{aligned} d_{A,B}^{y^{-},y,y^{+}}(r) = t_{r,A,y} * (t_{r,B,y^{+}} - t_{r,B,y^{-}}) - t_{r,B,y} * (t_{r,A,y^{+}} - t_{r,A,y^{-}}) \end{aligned}$$

A’s degree of being followed by B in year y considering the change of research topic from $y^-$ to $y^+$, $D_{A,B}^{y^-,y,y^+}$ was calculated from the sum of the $d_{A,B}^{y^-,y,y^+}(r)$ for the entire set of references $R_{all}$. We divided the values by their similarities for the entire set of references at y [denominator in Eq. (6)]. This is because the closer the distance between $\textbf{T}$ in the pair of nations, the closer the mutual relationship, and the easier it was to propagate the topic. Considering that the L2 norm of $\textbf{T}$ equals 1, $D_{A,B}^{y^-,y,y^+}$ was calculated as the basic arithmetic operation of the cosine similarity of $\textbf{T}$ [Eq. (7)]. Intuitively, the quantity $D_{A,B}^{y^-,y,y^+}$ is the difference between the amounts of rise of the red and blue lines in Fig. 5b divided by the cosine similarity of $\textbf{T}$ between the pair of nations at y.

6 $$\begin{aligned} D_{A,B}^{y,y^{-},y^{+}}&= \sum _{r \in R_{all}} d_{A,B}^{y^{-},y,y^{+}}(r) / \sum _{r \in R_{all}} t_{r,A,y} *t_{r,B,y} \end{aligned}$$

7 $$\begin{aligned}&= \dfrac{(cos(\textbf{T}_{A,y},\textbf{T}_{B,y^{+}})- cos(\textbf{T}_{A,y},\textbf{T}_{B,y^{-}}))- (cos(\textbf{T}_{B,y},\textbf{T}_{A,y^{+}}) - cos(\textbf{T}_{B,y},\textbf{T}_{A,y^{-}}))}{cos(\textbf{T}_{A,y}, \textbf{T}_{B,y})} \end{aligned}$$

Equation (8) describes the non-normalized TPI of nation A at y, $TPI_{A,y}^\prime$. We calculated averaged $D_{A,X,y}^{y^-,y,y^+}$ for all other nations weighted by the share of the number of published papers of nation X at year y, $S_X^y$. Then we summed the value for all $(y^-,y^+)=(y-1,y+1),\ldots ,(y-\tau ,y+\tau )$. We used $\tau = 5$ years to consider both short-term topic transitions, such as in computer science, and long-term transitions, such as in the humanities. When the similarity in research topics between A and B was low, $cos(\textbf{T}_{A,y},\textbf{T}_{B,y})<0.005$, and we considered $D_{A,B}^{y^-,y,y^+}=0$ to avoid large responses to small changes in the research topics of A or B. Finally, ${TPI}^\prime$ for each nation in a particular year was standardized such that the average was 0 and the standard deviation was 1. Consequently, we obtained the TPI [Eq. (9)]:

8 $$\begin{aligned} TPI^{\prime }_{A,y}&= \sum \limits _{X \ne A} \sum \limits _{\Delta y \in 1...\tau } D_{A,X}^{y-\Delta y,y,y+\Delta y} S_X^y \end{aligned}$$

9 $$\begin{aligned} TPI_{A,y}&= \frac{ TPI^{\prime }_{A,y} - \mu (TPI^{\prime }_{A^{\prime },y} \mid A^{\prime } \in nations) }{ \sigma (TPI^{\prime }_{A,y} \mid A^{\prime } \in nations)} \end{aligned}$$

Data limitations affected the calculation of TPI after $2020 - \tau$. When we calculated the TPI in 2019 with $\tau = 2$ years, the data for 2021 were missing. We assumed that the cosine similarity between $\textbf{T}_{y} \{y \mid y<=2020\}$ and $\textbf{T}_{y'} \{y' \mid y'>2020\}$ for any combination of nations were the same constant value. Consequently, when $y^+>2020$, $cos(\textbf{T}_{A,y},\textbf{T}_{B,y^+}$) and $cos(\textbf{T}_{B,y},\textbf{T}_{A,y^+})$ in Eq. (7) cancel each other. Thus, TPI after $2020-\tau$ is calculated from data up to 2020.

### Centrality analysis in the co-authorship network

We constructed a co-authorship network for 2018 from the preceding 20 years of co-author relationships. When N authors authored a paper, the weight of each edge was $1/(\hbox {N}-1)$, assuming that one author interacted equally with the remaining N-1 authors. Papers with more than 30 authors were ignored to avoid the impact of hyperauthorship. Furthermore, only the largest connected component was extracted for analysis. Eigenvector centrality (weighted), PageRank (weighted, $\alpha = 0.85$) and degree centrality (un-weighted) were calculated for each node using the igraph library^61^.

## Supplementary Information

Supplementary Information.

## Data Availability

The data that support the findings of this study are available from Elsevier but restrictions apply to the availability of these data, which were used under license for the current study, and so are not publicly available. Data are however available from the author (Kimitaka Asatani) upon reasonable request and with permission of Elsevier.
