# Supplementary material for: Quantifying progress in research topics across nations
Source: Sci Rep. 2023 Mar 23;13:4759. doi: 10.1038/s41598-023-31452-8 (PMC10036561; doi:10.1038/s41598-023-31452-8)
Supplement: Supplementary file 1 — Supplementary Information. [file 41598_2023_31452_MOESM1_ESM.pdf]

Supplementary Materials for
Quantifying progress in research topics across nations.

Kimitaka Asatani,* Sumihiro Oki, Takuya Momma, Ichiro Sakata

*Corresponding author. Email; asatani@tmi.t.u-tokyo.ac.jp.

This PDF file includes:
Figures. S1 to S10
Tables. S1, S2

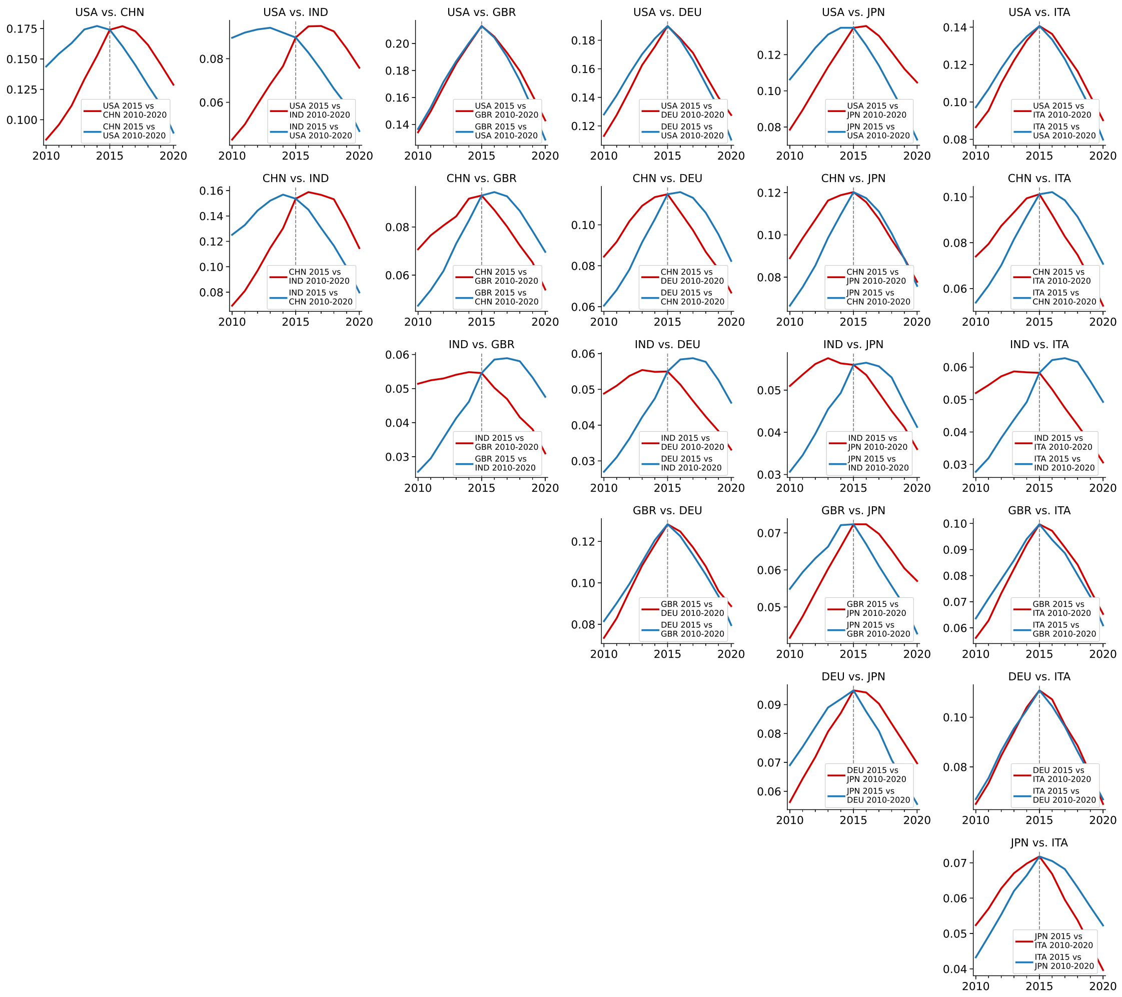

Fig S1. Time series comparison of research topic T among the top seven paper-publishing nations. Each panel compares nation A vs B. Red lines indicate cosine similarities between **T** in 2015 of nation A and **T** in 2010–2020 of nation B. Blue lines indicate the opposite comparisons.

Figures demonstrate the time lag in research topics between nations. In the case of US and the China, the positive slope of the red line around 2015 indicates that China followed US research topics in that year. The negative slope of the blue line around 2015 indicates that the China followed US in that year. The difference in the slopes between the blue and red line indicates that China follows the US in research topics. Japan shows the same pattern as China in terms of a delayed following of the US. Other graphs also indicate leading–following relationships between pairs of nations. For instance, India and Russia are significantly behind the other five nations in research topics. Slight delays in the research topic of the UK, Germany, and Italy to the US are observed.

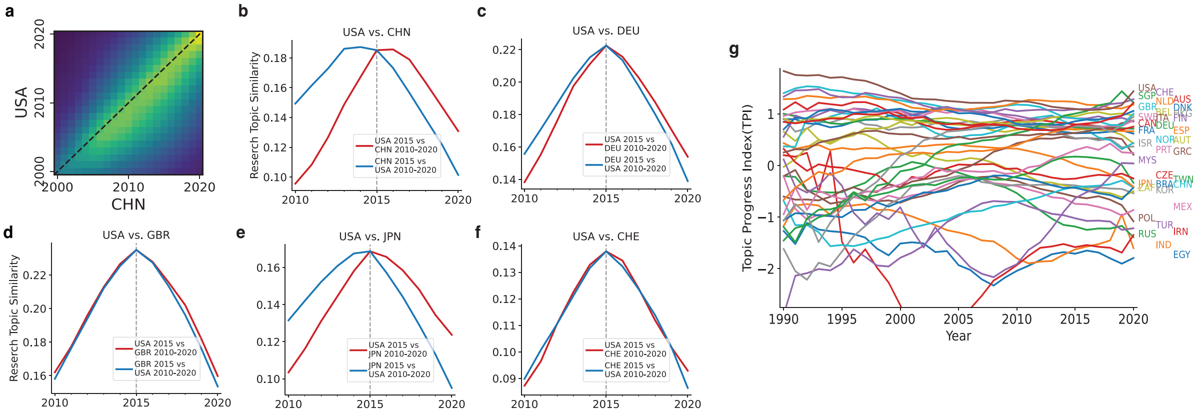
a
USA
CHN
2000
2010
2020
b
USA vs. CHN
Reserch Topic Similarity
USA 2015 vs CHN 2010-2020
CHN 2015 vs USA 2010-2020
c
USA vs. DEU
USA 2015 vs DEU 2010-2020
DEU 2015 vs USA 2010-2020
d
USA vs. GBR
Reserch Topic Similarity
USA 2015 vs GBR 2010-2020
GBR 2015 vs USA 2010-2020
e
USA vs. JPN
USA 2015 vs JPN 2010-2020
JPN 2015 vs USA 2010-2020
f
USA vs. CHE
USA 2015 vs CHE 2010-2020
CHE 2015 vs USA 2010-2020
g
Topic Progress Index(TPI)
Year
1990
1995
2000
2005
2010
2015
2020

Fig S2. Research topic comparison and TPI using fractional counting (a) The cosine similarity matrix of $\mathbf{T}$ between China and the US from 2000 to 2020.**(b-f)** Two-nation comparisons: red lines indicate cosine similarities between $\mathbf{T}$ in 2015 in the US and $\mathbf{T}$ in 2010–2020 in China **(b)**, Germany **(c)**, the UK **(d)**, Japan **(e)**, and Switzerland **(f)**. Blue lines indicate the opposite comparisons (in the other nation in 2015 and in the US in 2010–2020). **(g)** Yearly change in the TPI of the top 40 paper-publishing nations from 1990 to 2020.

Figures show the topic comparison between nations using fractional counting. When we use fractional counting for author assignment to nation(s), two nations with international co-authorship papers tend to have a high similarity in research topics in the same year. Therefore, international co-authored papers between two nations are excluded when calculating the research topic similarity between them. The results using fractional counting show the same trend as those obtained from assigning papers to first authors' nations, as depicted in Figure 1. Compared to first author assignment, the US's significant progress among Western nations is not observed with fractional counting. This can be due to a high number of international co-authorship between the Western nations.

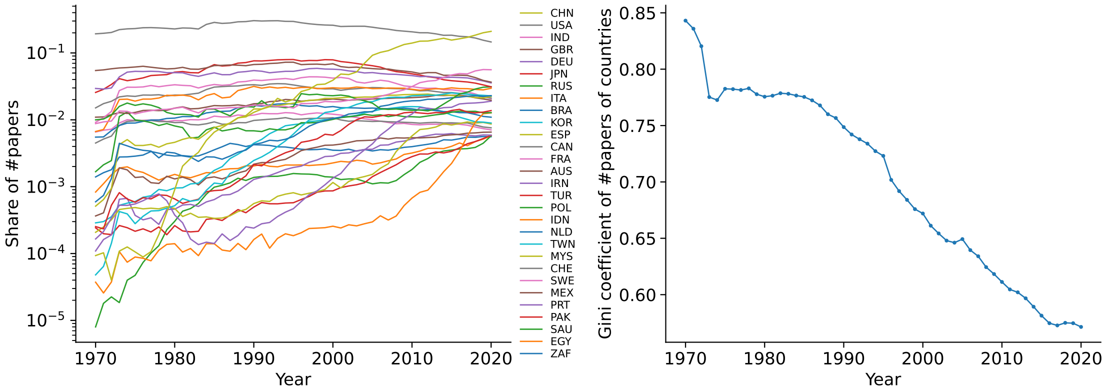

Fig S3. Time series of the share and dispersion of published papers by nations. (left) Trends in the share of papers (log scale) among the top 30 nations. China and India significantly increased their share of published papers, as did other developing nations. (right) Annual change in the Gini coefficient of the share of papers in all 207 nations.

Figures present the shrinking gap between nations in the number of published papers. Most nations that had published many papers before, such as the US and UK, had a lower share of papers published in recent decades. The left figure indicates that some nations, such as China and India, increased their share of papers published in recent decades. The change in the Gini coefficient of the share of the number of papers over time shown in the right figure also shows a shrinking gap in the number of papers published.

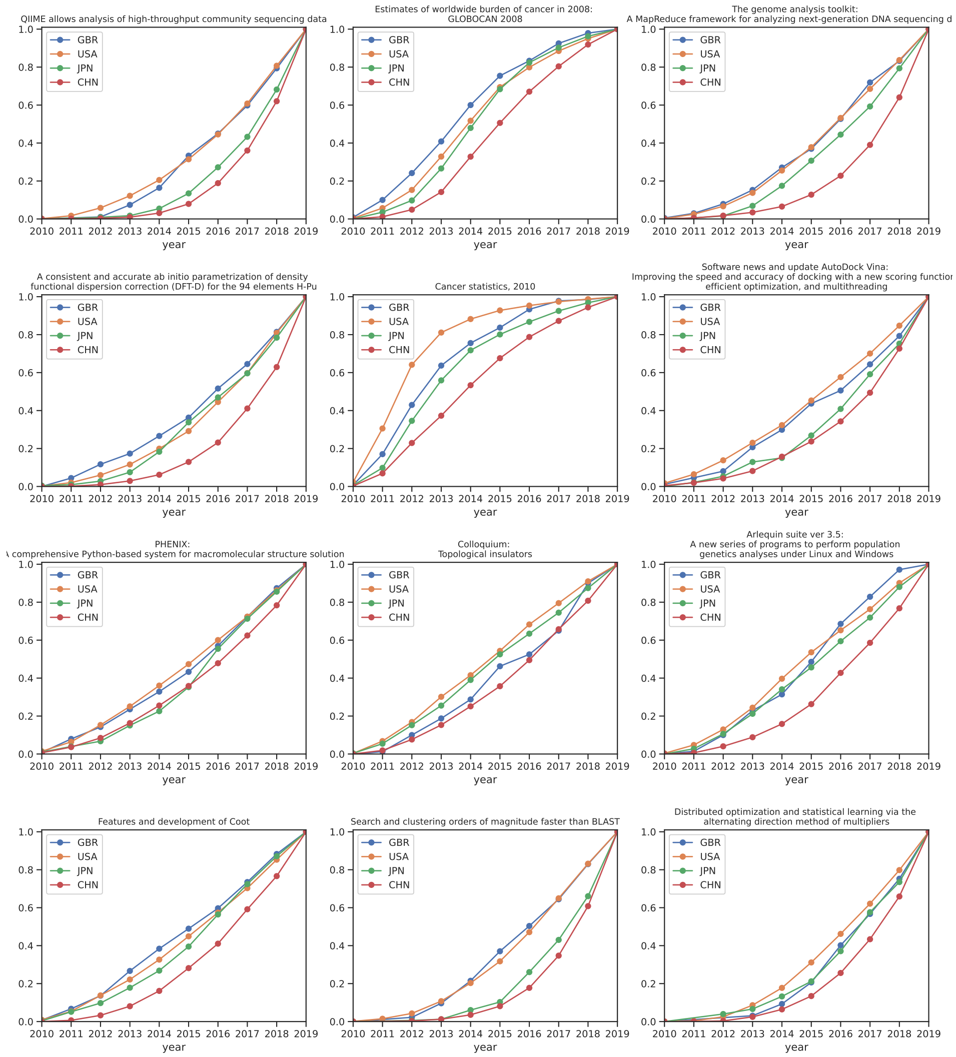

Fig S4. Examples of top-cited papers' citation evolution for each nation Each figure shows the increase in the number of citations for the US, UK, China, and Japan among the top 12 cited papers in 2010. The vertical axis indicates the cumulative number of papers that cite the target paper in a year divided by the same number for 2019.

Progress and delays in citations of top-cited papers between nations are observed in each nation's evolution of citing high-impact papers. Each figure shows the growth of citations in the US, the UK, China, and Japan in the top 12 highest-cited papers published in 2010. In some cases, such as the top-left figure, US and UK papers cite an article earlier than do papers from China and Japan. However, the opposite case is rare. This indicates that the US and UK engaged in important research topics earlier than China and Japan.

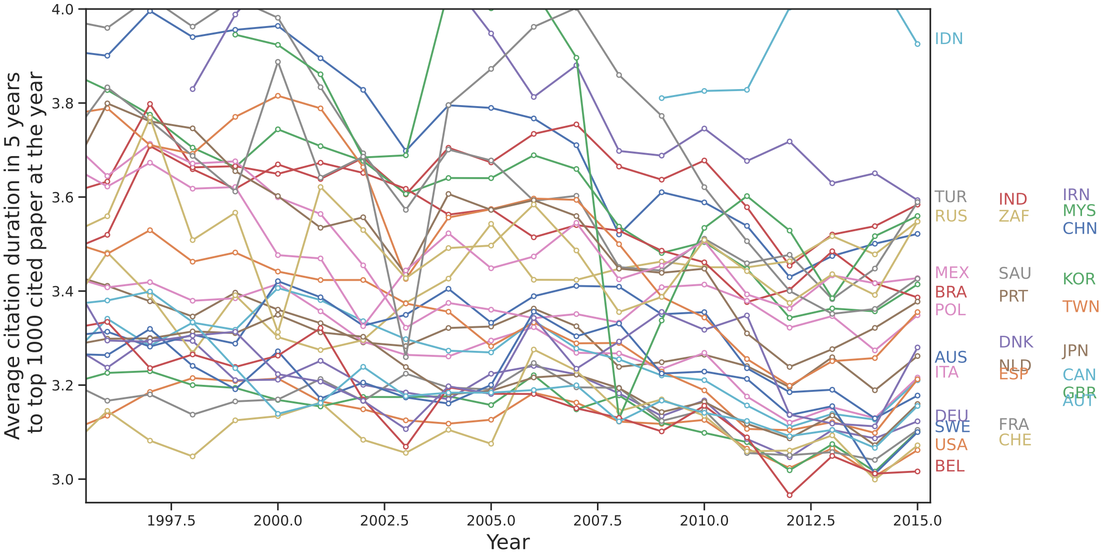

Fig S5. Each nation's initial citation speed to the top 1000 cited papers The figure shows each nation's time series of initial citation speed of the top 1000 cited papers published in a given year. Initial citation speed is the average citation duration(the difference between citing paper's publication year and cited paper's publication year) to future top 1000 cited papers in a given year from papers published in the next 5 years.

Assessment of initial citation speed to future top-cited papers would be a naïve method for assessing the progress of a research topic in a nation. This indicator, as shown in the figure, provides the same trend as the TPI: the speed for citations in India, Russia, and China is slow. However, the indicator is not robust: the leading nation changes annually. In addition, this indicator is biased toward the most-cited articles, and progress on a research topic is not evaluated until several(5) years later, when a certain number of citations have been published.

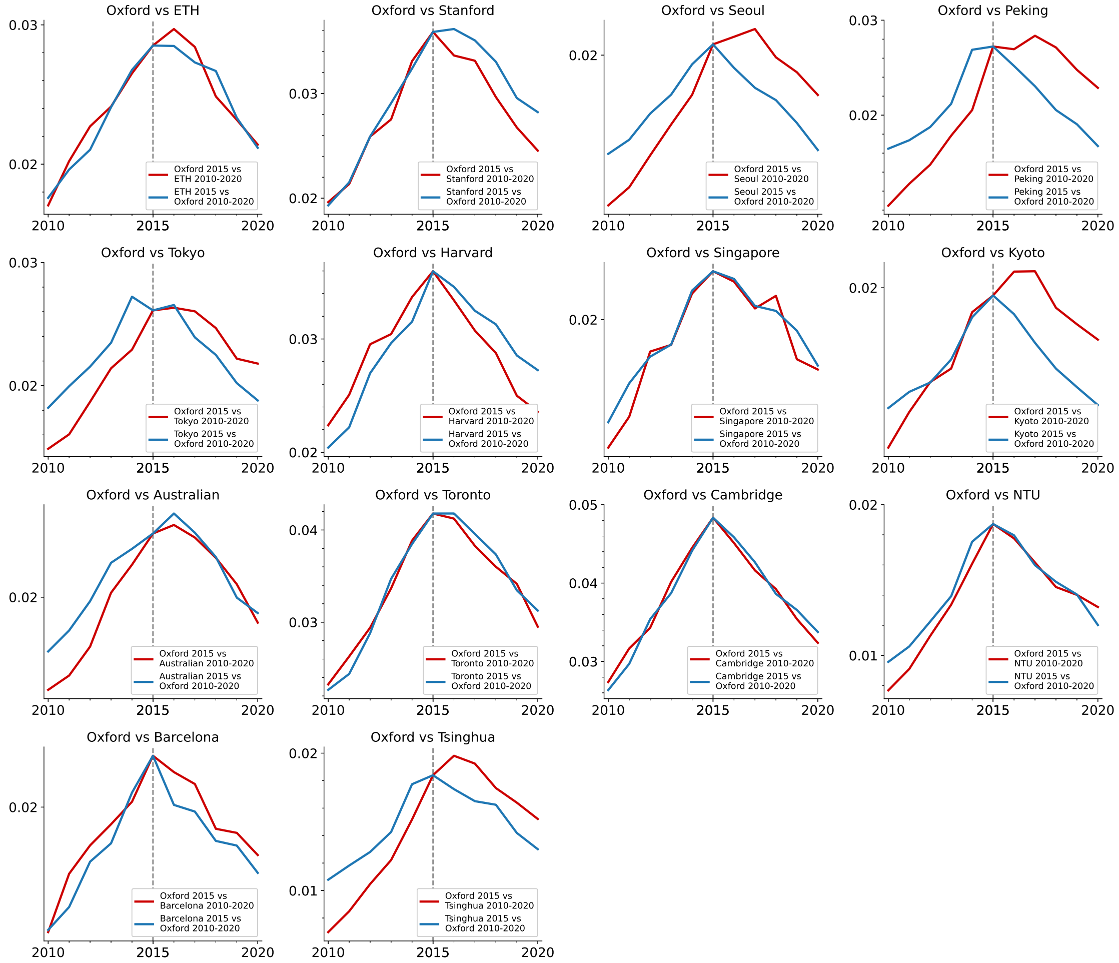

Fig S6. Time series comparison of research topic $\mathbf{T}$ between universities The figures display a comparison of the $\mathbf{T}$ progress and delay in research topics of several nationally representative universities versus Oxford University, which is ranked as the top university in the world. The red line in each figure (for the comparison of Oxford vs university A) indicates Oxford's 2015 research topics $\mathbf{T}_{\text{Oxford},2015}$ versus A's 2010–2020 research topics $\mathbf{T}_{A,2010}..\mathbf{T}_{A,2020}$. The blue line indicates A's 2015 research topics versus Oxford's 2010–2020 research topics.

We found that some universities such as Peking University (China) and Seoul (Korea) were behind Cambridge in research topics. However, no or a little delay or advance by Oxford University is apparent compared to Toronto, Harvard, Stanford, ETH, and National university of Singapore. These results match the nation-level comparison for the research topics $\mathbf{T}$ shown in Fig.1 (h).

S1-Social Science/ Humanities

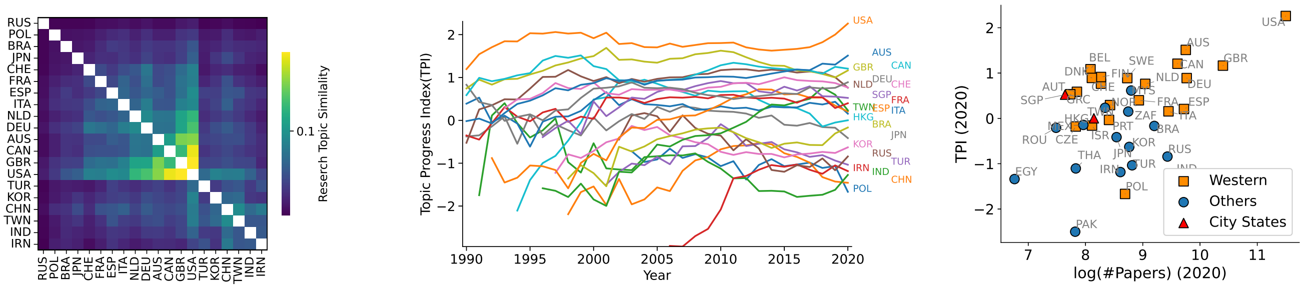

C1-Computer Science

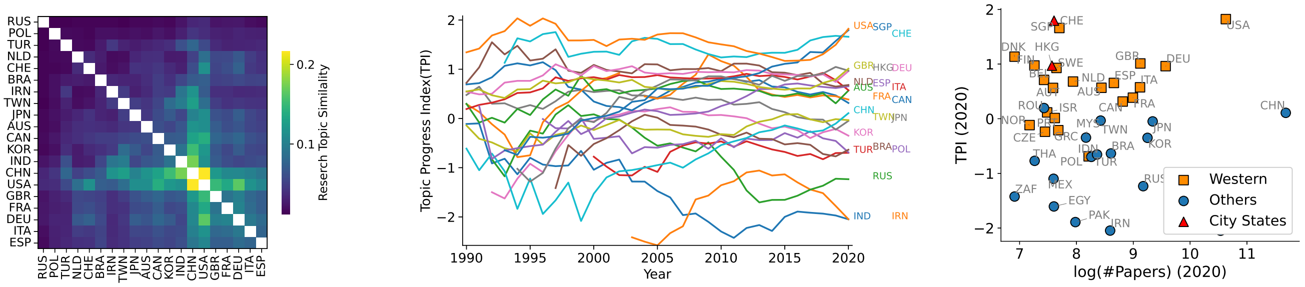

M1-Neuroscience

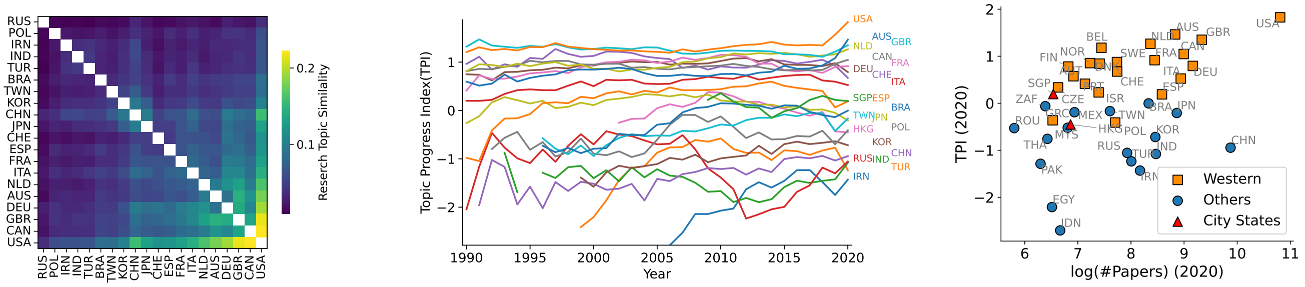

M2-Cancer

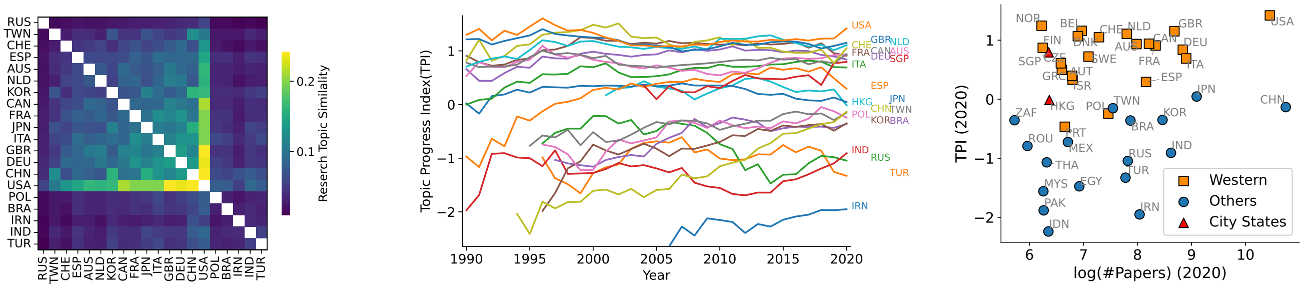

M3-Lifestyle Disease

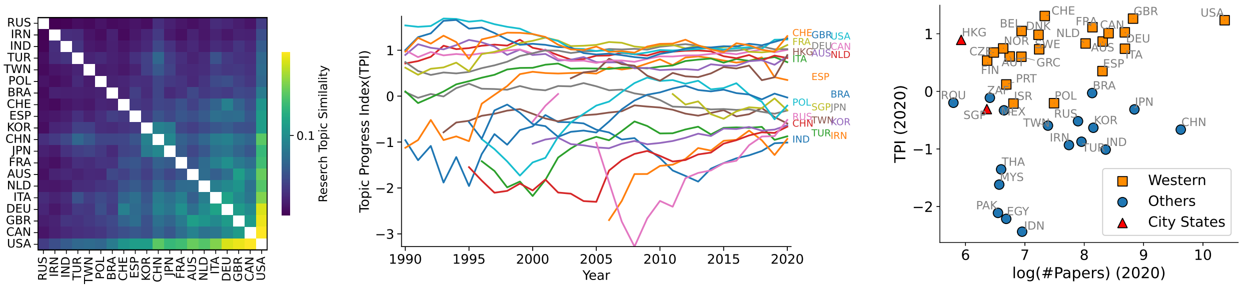

M4-Infectious Disease

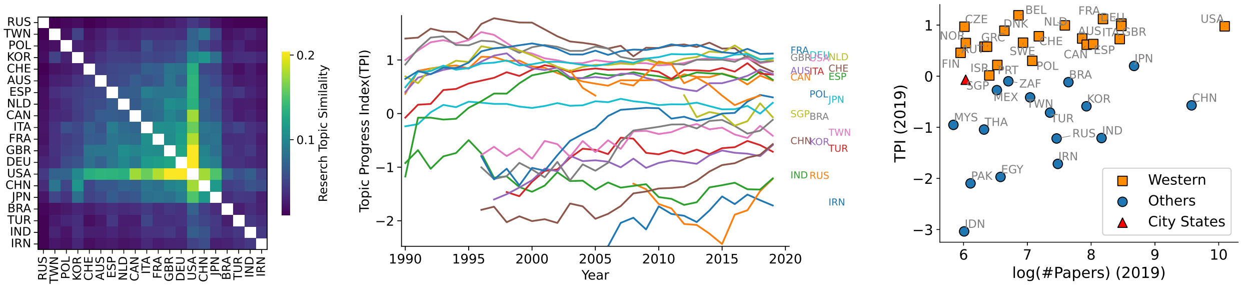

M5-Physiology

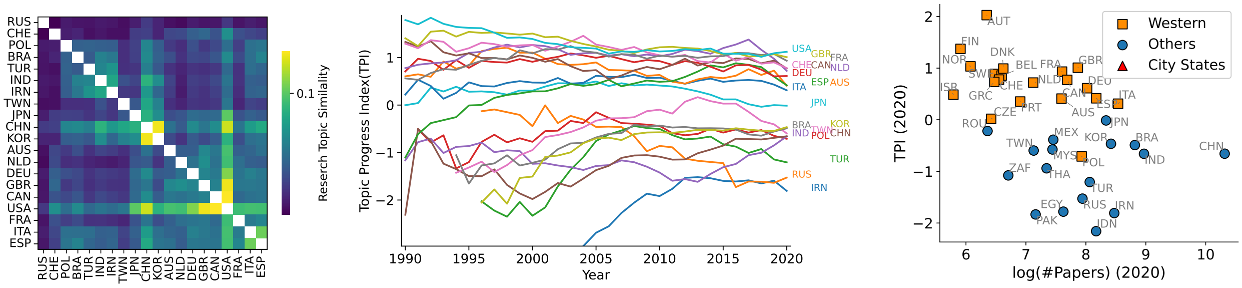

M6-Orthopedic/ Dentistry

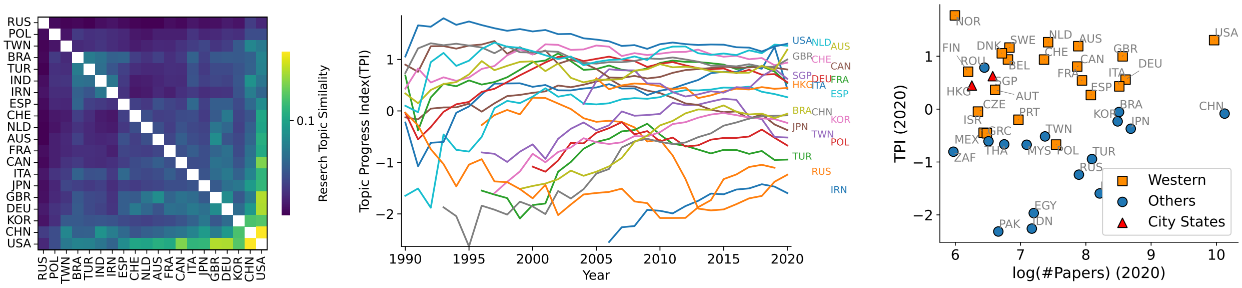

M7-Genetics

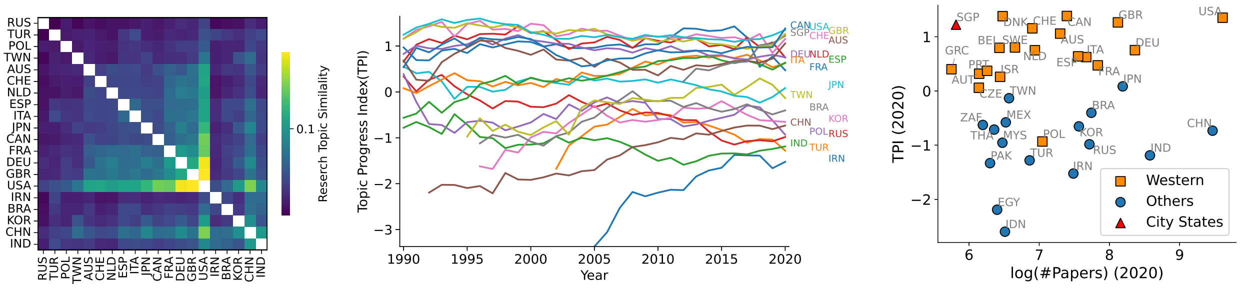

M8-Surgery

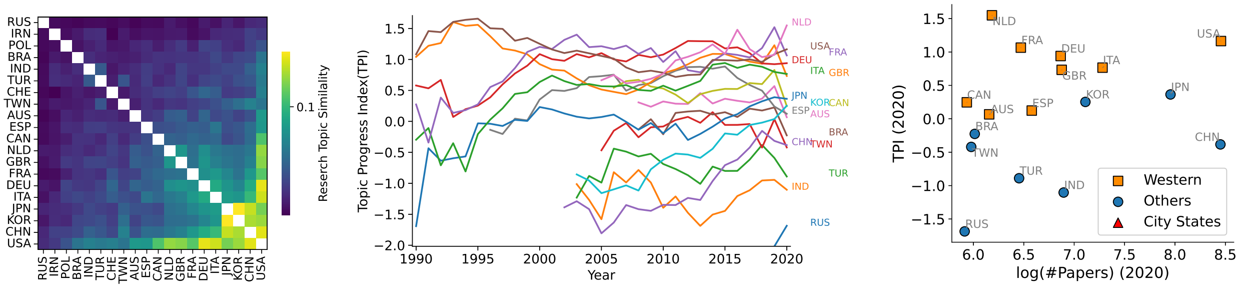

P1-Applied Physics

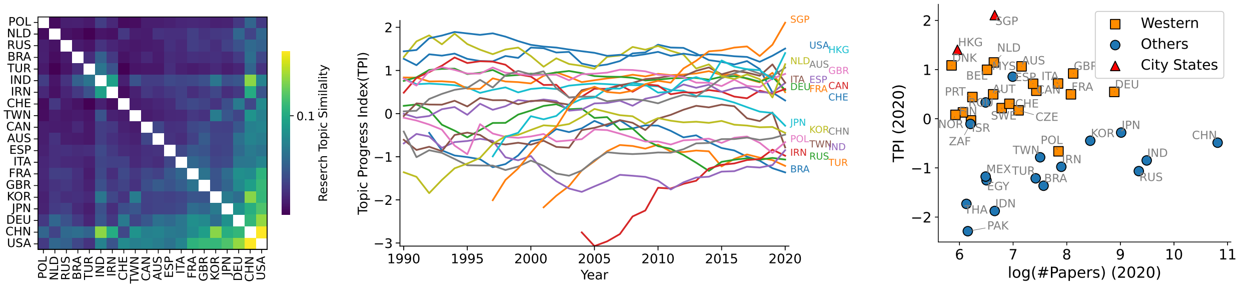

P2-Astronomy

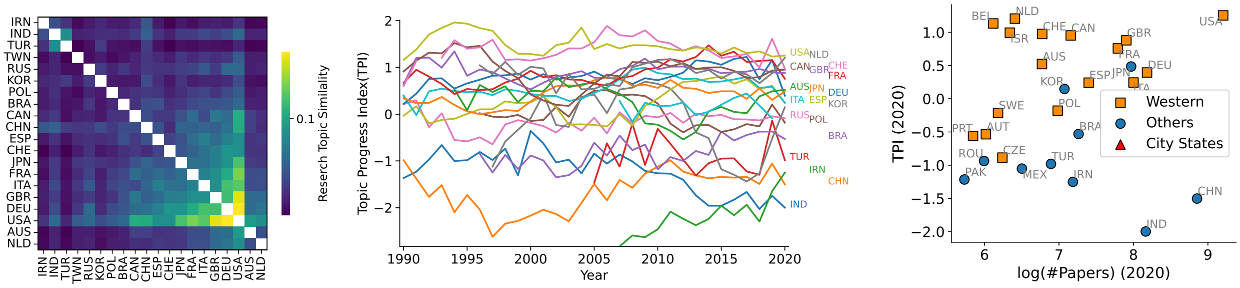

P3-High Energy Physics

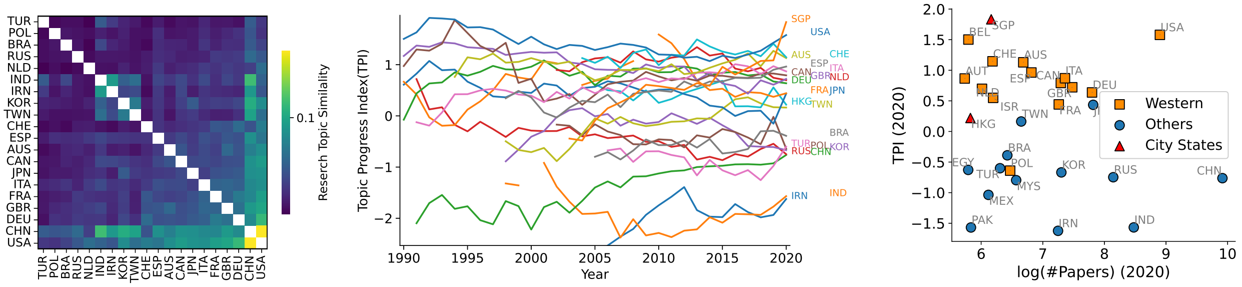

C1-Inorganic Chemistry

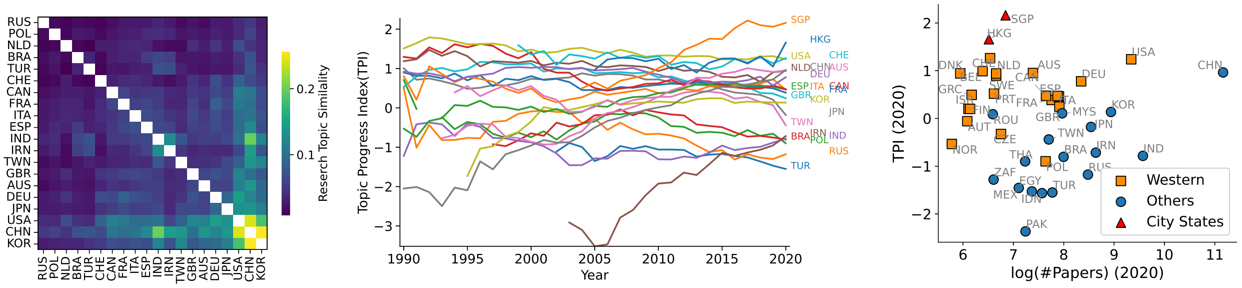

C2-Organic Chemistry

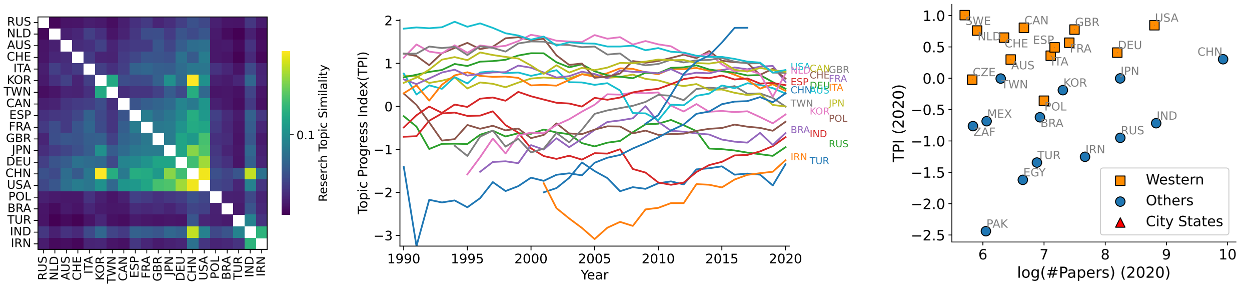

E1-Biological Sciences

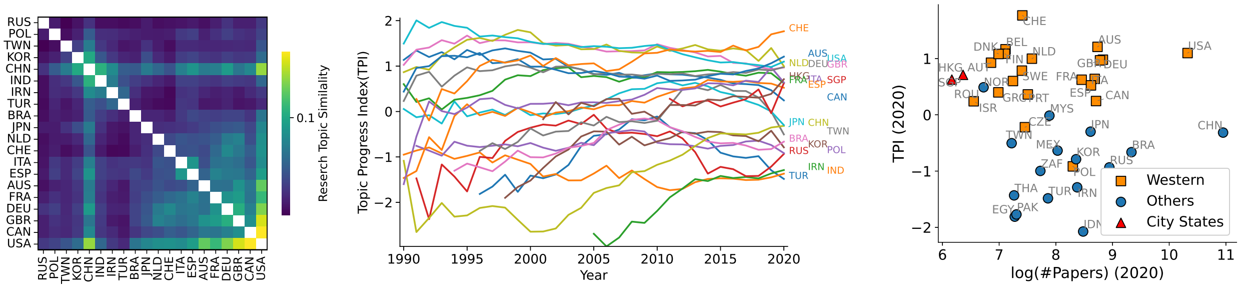

E2-Civil Engineering

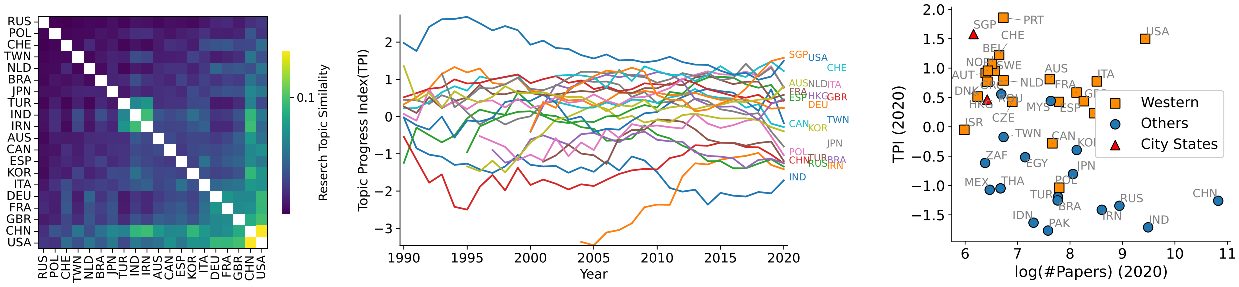

E3-Geology

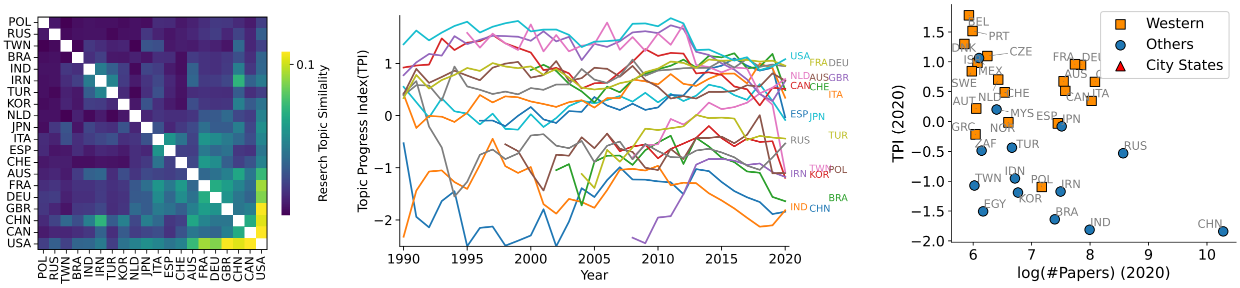

E4-Agriculture

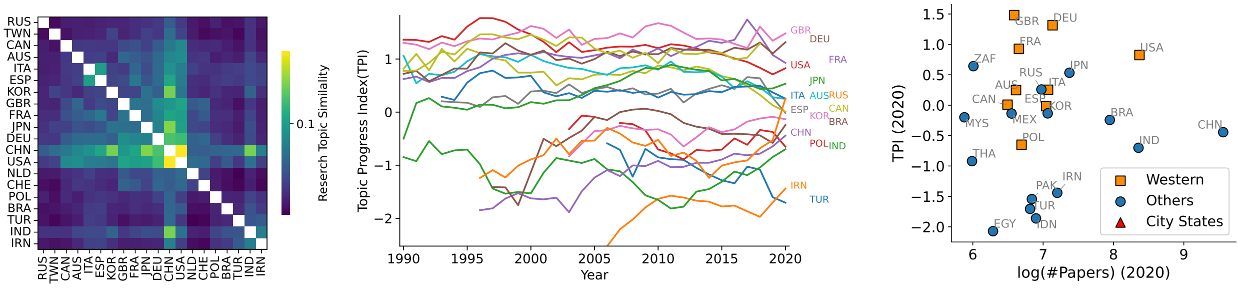

E5-Energy

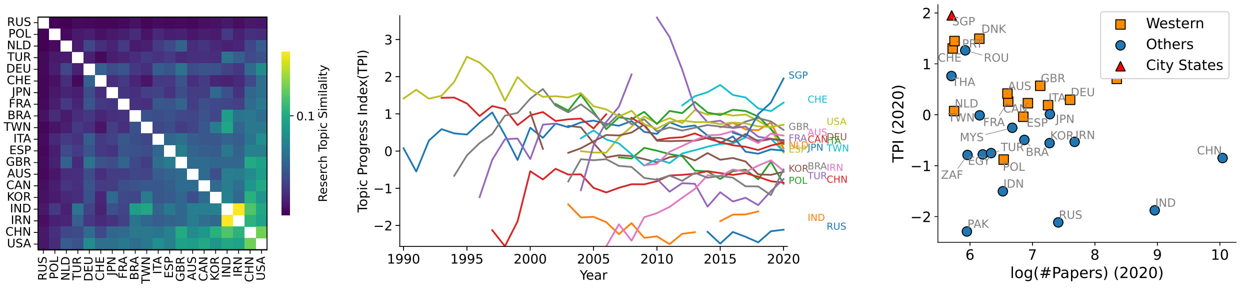

Fig S7. Detailed analysis of each domain Each figure shows a comparison of research topics $\mathbf{T}$ between nations for each domain, using information obtained from citation network clustering (as detailed in the Methods section and Supplemental Table T1). The left panel shows the cosine similarity matrix for research topic $\mathbf{T}$ between the top 30 nations in the number of papers in 2020, sorted by average linkage clustering. The middle figure shows the time series of TPI for the top 20 nations, plus Hong Kong and Singapore, from 1990 to 2020. The right panel presents scatterplots of TPI values of nations versus the number of papers for each domain. The TPI values are not plotted for nations with fewer than 300 papers published in a given year, as doing so would produce highly distorted values.

To summarize the results, we found that in the Western World, the research topics progressed in every cluster (middle panel), at least to some degree. In every cluster, the degree of the belonging core of similar research topics $\mathbf{T}$ (left panel) and the number of papers (x-axis in the right figure) do not sufficiently explain the topic progress of nations. The left panel indicates the existence of the core(s) of a similar research topic $\mathbf{T}$ in each field. In every cluster, the US belongs to the core.

We categorized the similarity matrix of research topic into three types: 1. Single Western World core, 2. Single Western World core with weak Asian core, and 3. Western World and Asian merged core. M1-Neuroscience, M3-Lifestyle Diseases, M4-Infectious Diseases, E3-Geology, and P2-Astronomy are all categorized as "Single Western World core". The Asian nations of China, Japan, and South Korea are not among the core nations on these research topics. Most of the peripheral nations have not advanced in research topics, with some exceptions (for example, South Korea shows advancement in 15-Astronomy).

Some clusters (S1-Social Science/Humanities, E1-Biological Sciences, M5-Physiology, and M7-Genetics) are categorized as "Single Western World core with weak Asian core". The nations at the core of the main research topic are mostly Western and Asian nations, pursuing their own similar research topics. Asian nations are found in the core likely because of the context-dependent research topic of these clusters. For example, urban problems, social issues, biocenosis, and biological environments differ across different regions. Despite regional differences in research purpose, there is a noticeable difference in progress in research topics between nations in the main core and the weak secondary core, indicating that the research topic is unilaterally disseminated from the main core nations to the weak secondary core nations.

In the clusters CS1-Computer Science, P1-Applied Physics, M2-Cancer, E2-Civil Engineering, M6-Orthopedics/Dentistry, C1-Inorganic Chemistry, C2-Organic Chemistry, P3-HighEnergy Physics, E4-Agriculture, and E5-Energy, Asian nations (mostly China, Japan, and South Korea) are also located at research topic cores together with Western nations ("Western World and Asian merged core"). However, these Asian nations do not show advancement in these topics. For example, in cluster 1, China takes a central position both in the research topic and the number of papers, but its TPI does not show a high value. This indicates that conducting large amounts of research on topics where the US has leadership does not necessarily lead to progress in such topics.

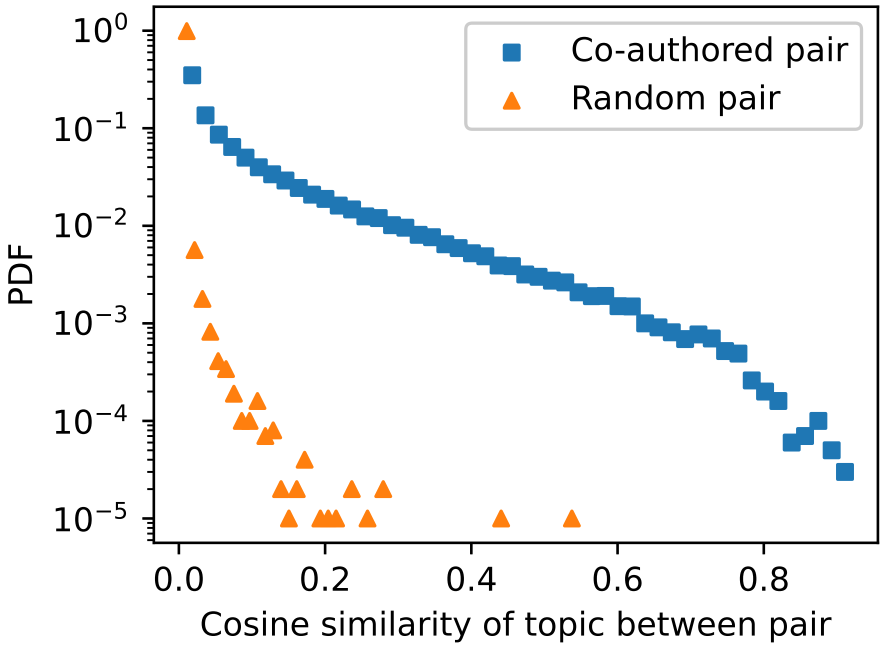

Fig S8. Research topic T similarity for authors who have co-authored and for random author pairs Probability distribution of the cosine similarity for research topics $\mathbf{T}$ among all publications between pairs of scientists (except for co-authored publications). The orange histogram shows the similarity of research topic $\mathbf{T}$ between randomly chosen author pairs. The blue histogram indicates the relationship between pairs who have been co-authors more than once.For better comparison, we analyzed authors who had published more than 100 papers.

Not surprisingly, the research topic $\mathbf{T}$ similarity between co-authored pairs (blue) was higher than that between the randomly chosen pairs (orange). This indicates that research topics are disseminated on coauthorship networks.

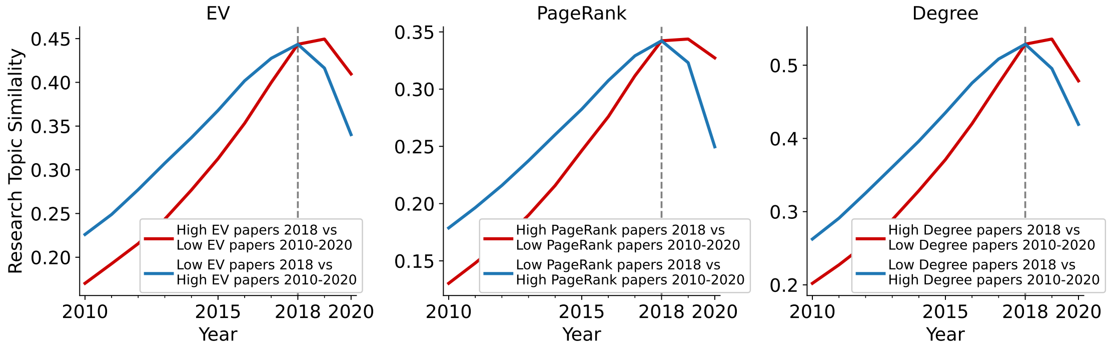

Fig S9. Research topic progress of the top 50% of papers in author centrality Figures present comparisons by research topic $\mathbf{T}$ of the top 50% of papers based on highest-author-centrality (Eigenvector Centrality (EV), left; PageRank, middle; Degree Centrality, right) and low highest-author-centrality papers. The red line in each panel (high author centrality papers vs. others) indicates the high author centrality papers' 2018 research topic $\mathbf{T}$ and low author centrality papers' 2010–2020 research topic $\mathbf{T}$. The blue line indicates the low author centrality papers' 2018 research topic $\mathbf{T}$ and high author centrality papers' 2010–2020 research topic $\mathbf{T}$.

We found that papers whose authors have high centrality show advancement in a topic relative to other papers. Differences between measures of centrality (Degree, Eigenvector Centrality (EV), PageRank) are not clearly observed.

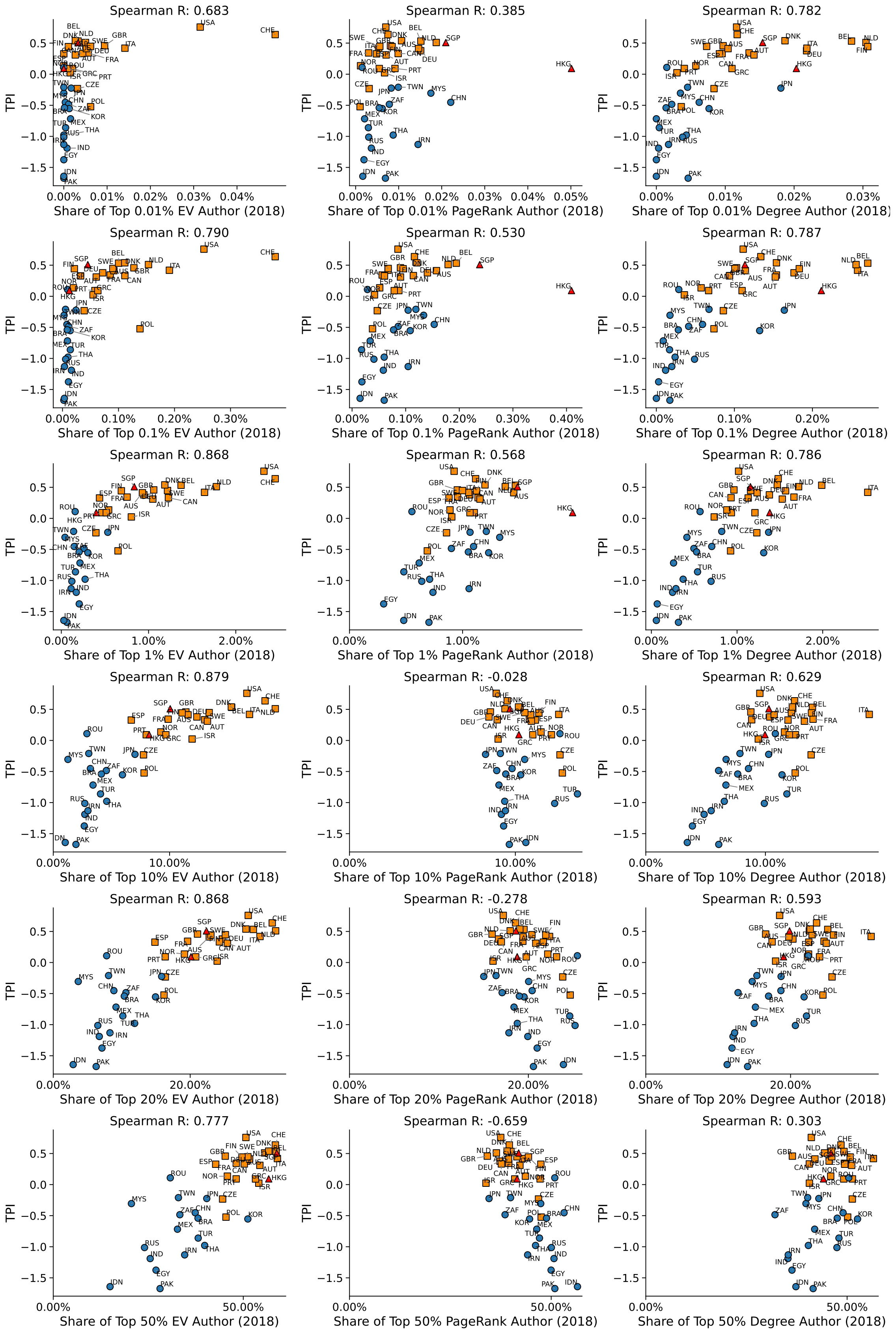
Spearman R: 0.683
Share of Top 0.01% EV Author (2018)
Spearman R: 0.385
Share of Top 0.01% PageRank Author (2018)
Spearman R: 0.782
Share of Top 0.01% Degree Author (2018)
Spearman R: 0.790
Share of Top 0.1% EV Author (2018)
Spearman R: 0.530
Share of Top 0.1% PageRank Author (2018)
Spearman R: 0.787
Share of Top 0.1% Degree Author (2018)
Spearman R: 0.868
Share of Top 1% EV Author (2018)
Spearman R: 0.568
Share of Top 1% PageRank Author (2018)
Spearman R: 0.786
Share of Top 1% Degree Author (2018)
Spearman R: 0.879
Share of Top 10% EV Author (2018)
Spearman R: -0.028
Share of Top 10% PageRank Author (2018)
Spearman R: 0.629
Share of Top 10% Degree Author (2018)
Spearman R: 0.868
Share of Top 20% EV Author (2018)
Spearman R: -0.278
Share of Top 20% PageRank Author (2018)
Spearman R: 0.593
Share of Top 20% Degree Author (2018)
Spearman R: 0.777
Share of Top 50% EV Author (2018)
Spearman R: -0.659
Share of Top 50% PageRank Author (2018)
Spearman R: 0.303
Share of Top 50% Degree Author (2018)
TPI

Fig S10. Relationship between TPI and the proportion of high network centrality authors in each nation The figure shows the relationship between TPI and the proportion of top (0.01%, 0.1%, 1%, 10%, 20%, 50%) network centrality (eigenvector centrality (EV), left; PageRank, center; and degree, right) authors in each nation's co-authorship network.

The fourth figure from the top on the left shows the strongest correlation between TPI and the proportion of top 10% eigenvector centrality authors. These results indicate that the percentage of authors who belong to the core of global information flow relates to the advancement of the topic. However, we do not find such strong correlations between TPI for each nation and the proportion of top PageRank or degree authors .

Table T1. Details of domains obtained by citation-network clustering using the Leiden method Domains are obtained by citation-network clustering using the Leiden method. Each domain(cluster) is named by authors based on the top tfidf terms and randomly sampled papers from it. The top eight tfidf terms are displayed after 100,000 samples of documents for each domain. We also conducted recursive clustering and obtained sub-domains. We show the top two tfidf terms for each sub-domains in the table.

Domains consisting of more than 500,000 papers are analyzed in this paper. We categorized the domains into the following six groups. In each group, domains were ordered by the number of papers within them.

- Social Science/Humanities: S1-Social Science/Humanities
- Computer Science: CS1-Computer Science
- Medical Science: M1-Neuroscience, M2-Cancer, M3-Lifestyle Diseases, M4-Infectious Diseases, M5-Physiology, M6-Orthopedics/Dentistry, M7-Genetics, M8-Surgery
- Physics: P1-Applied Physics, P2-Astronomy, P3-High-Energy Physics
- Chemistry: C1-Inorganic Chemistry, C2-Organic Chemistry
- Environmental Science and Civil Engineering: E1-Biological Sciences, E2-Civil Engineering, E3-Geology, E4-Agriculture, E5-Energy

S1-Social Science/Humanities	7,668,640
Social, Political, Education, Public, Role, Child, Organizational, Effect	
0. Political, Politic, 1. Teacher, Student, 2. Medical, Physician, 3. Online, Social, 4. Monetary, Financial, 5. Social, Crime, 6. Nineteenth, Moral, 7. Tourism, Cultural, 8. Organizational, Entrepreneurial, 9. Sustainable, Pay, 10. Fuzzy, Time window, 11. Organizational, Job satisfaction, 12. Urban, Social, 13. Lesbian, Sexual, 14. Social, Emotion, 15. Accident, Occupational, 16. Autism, Disability,	

CS1-Computer Science	6,013,703
Application, Wireless sensor network, Adaptive, Design, Semantic, Mobile, Algorithm, System	

0. Robust, Nonlinear, 1. UML, Object, 2. LTE, OFDM, 3. Wireless sensor network, IEEE, 4. Fuzzy, Neural, 5. Robot, Mobile robot, 6. Semantic, Face recognition, 7. Image, Visual, 8. Image segmentation, Change detection, 9. Semantic, Ontology, 10. Reconfigurable, Cache, 11. Peer, H., 12. Web service, Cloud, 13. Signal, Adaptive, 14. Privacy, Blockchain, 15. Graph, Planar graph, 16. Bayesian, Random vector, 17. Satellite, Lunar, 18. Robust speech recognition, Speech enhancement, 19. Chaotic, Chao, 20. Fault detection, Fault diagnosi, 21. Ant, Ant colony optimization, 22. Complex network, Epidemic, 23. Haptic, Virtual,

M1-Neuroscience	4,313,716
Effect, Patient, Rat, Cognitive, Schizophrenium, Child, Disease, Alzheimer	
0. Visual, Cognitive, 1. Depression, Borderline personality disorder, 2. Alzheimer, Dementium, 3. Synaptic, Nicotinic, 4. Vestibular, Stroke, 5. Parkinson, Disease, 6. Schizophrenium, Schizophrenic, 7. Rat, Cocaine, 8. Cochlear, Cochlear implant, 9. Cigarette, Methadone, 10. Migraine, Neuropathic, 11. Epidural, Dexmedetomidine, 12. Antidepressant, Depression, 13. Oxytocin, Neuropeptide, 14. Palliative, Life, 15. Sleep, Circadian, 16. Spinal, Neurotrophic, 17. Epilepsy, Epileptic, 18. Traumatic, Traumatic brain injury, 19. ADHD, Autism, 20. Lexical, English,	

M2-Cancer	3,614,850
Patient, Human, Clinical, Expression, Effect, Breast cancer, Ovarian, Prostate cancer	
0. Sperm, Oocyte, 1. Apoptosi, Autophagy, 2. MicroRNA, microRNA, 3. Breast cancer, Breast, 4. Zebrafish, Caenorhabditis elegan, 5. Chromatin, Epigenetic, 6. Endometriosi, Endometrial, 7. Kinase, Protein, 8. Prostate cancer, Prostate, 9. Fanconi, Topoisomerase, 10. Chromosome, Trisomy, 11. Gastrointestinal stromal tumor, Gastrointestinal stromal, 12. Small cell lung cancer, Lung cancer, 13. Thyroid, Thyroid carcinoma, 14. Myelodysplastic, Acute myeloid leukemium, 15. HIF, MMP, 16. Yeast, Proteomic, 17. Malignant melanoma, Merkel cell carcinoma, 18. Head, Neck cancer, 19. Meningioma, Glioblastoma, 20. Ribosomal, Actinomycin, 21. Renal, Metastatic renal cell carcinoma, 22. Pituitary, Acromegaly, 23. Cervical, Cervical cancer, 24. Stem cell, Embryonic, 25. Irinotecan, Metastatic colorectal cancer, 26. Mitotic, Microtubule, 27. Metaplastic, Epidermolysis bullosa simplex,	

M3-Lifestyle Disease	3,364,246
Patient, Effect, Cardiac, Coronary, Clinical, Diabete, Cardiovascular, Ventricular	

0. Fetal, Preterm, 1. Obesity, Overweight, 2. Hypertension, Antihypertensive, 3. Venou, Von, 4. Coronary, Acute myocardial infarction, 5. Extracorporeal, Sepsi, 6. Mitral, Aortic, 7. Acute ischemic, Acute ischemic stroke, 8. Angiotensin Ius, Angiotensin, 9. Ventricular, Cardiac, 10. Diabete, Type, 11. Endothelin, Pulmonary, 12. Heart failure, Atrial natriuretic, 13. Intubation, Endotracheal, 14. Aortic, Endovascular, 15. Cardiopulmonary, Cardiopulmonary bypas, 16. Renal, Renal artery stenosi, 17. Coronary, Lipid, 18. Atrial fibrillation, Atrial, 19. Heart rate variability, Syncope, 20. Left atrial myxoma, Left atrial, 21. Obstructive sleep apnea, Obstructive, 22. Thyroid, Iodine, 23. Arterial, Treat, 24. Clinical, Clinical trial,

M4-Infectious Disease	2,988,562
Patient, Human, Interleukin, Clinical, IL, HIV, Effect, Treatment	
0. Asthma, Allergic, 1. Lymphocyte, Thymu, 2. IL, Interleukin, 3. HIV, Antiretroviral, 4. Influenza, H1N1, 5. Hepatitis viru, Hepatiti, 6. Tuberculosi, Mycobacterium tuberculosi, 7. Simplex, Kaposi, 8. Rheumatoid arthriti, Psoriasi, 9. Systemic lupus erythematosu, Systemic, 10. Lymphoma, Chronic lymphocytic, 11. Multiple sclerosi, Chronic inflammatory, 12. Dendritic cell, Immune, 13. Human, Oligosaccharide, 14. Chronic obstructive pulmonary, Cystic, 15. Graft versus host disease, Hematopoietic, 16. Wegener, Granulomatosi, 17. Crohn, Ulcerative, 18. Enteroviru, Enteroviruse, 19. COVID, Pandemic, 20. Sarcoidosi, Idiopathic pulmonary fibrosi, 21. Rejection, Renal, 22. Complement, Complement factor, 23. Toxic epidermal necrolysi, Allergic, 24. Multiple myeloma, Relapsed, 25. Celiac, Celiac disease, 26. Type, Diabete, 27. Retroviral, Oncolytic,	

M5-Physiology	2,972,195
Effect, Rat, Human, Antioxidant, Dietary, Diet, Oxidative, Patient	
0. Flavonoid, Essential oil, 1. Cytochrome, Mutagenicity, 2. Physicochemical, Effect, 3. Glucose, Adipose, 4. Oxidative, Vitamin, 5. Dairy cow, Ruminal, 6. Probiotic, Gut microbiotum, 7. Arsenic, Lead, 8. Skeletal, Cachexium, 9. Lipoprotein, Apolipoprotein, 10. Olive, Wine, 11. Fatty acid, Fatty, 12. Pesticide, Bisphenol, 13. Mitochondrial, Myocardial, 14. Gastric, Cyclooxygenase, 15. Carnitine, McArdle, 16. Vitamin, Thiamine, 17. Complementary, Aristolochic, 18. Polysaccharide, Ganoderma lucidum, 19. Metabolomic, Methoxy, 20. Nrf2, Heme oxygenase, 21. Polycystic ovary, Polycystic ovary syndrome, 22. Carbohydrate deficient transferrin, Hepatic, 23. Acne, Acne vulgari,	

M6-Orthopedic/Dentistry	2,643,315
Effect, Patient, Clinical, Treatment, Poly, Dental, Vitro, Case	

0. Mesenchymal, Osteogenic, 1. Osteoporosi, Bone, 2. Liposome, Poly, 3. Pain, Cervical, 4. Ankle, Patellar, 5. Femoral, Total hip arthroplasty, 6. Periodontal, Periodontal disease, 7. Temporomandibular, Orthodontic, 8. Endodontic, Dental, 9. Articular, Cartilage, 10. Flap, Microneurovascular, 11. Styrene, Poly, 12. Chitosan, Hydrogel, 13. Capillary, Microfluidic, 14. Photoacoustic, Photodynamic, 15. Lymphedema, Chronic venou, 16. Carpal, Carpal tunnel syndrome, 17. Humeral, Shoulder, 18. Mml, Formulation, 19. Vertebroplasty, Aneurysmal bone cyst, 20. Transdermal, Skin,

M7-Genetics	2,405,233
Molecular, Escherichia coli, Protein, Effect, Antibiotic, Characterization, High, Antimicrobial	
0. Antibiotic, Resistant, 1. Lipase, Enzymatic, 2. HFC, Binary, 3. Escherichia coli, Pseudomonas aeruginosa, 4. Protein, Molecular, 5. Salmonella, Listeria monocytogene, 6. DNA, Atomic force microscopy, 7. Pneumonium, Meningococcal, 8. Cytochrome, Hemoglobin, 9. Lipid, Lipid bilayer, 10. Surfactant, Asphaltene, 11. tRNA, Ribosomal, 12. Chromatographic, Stationary phase, 13. Bacteriorhodopsin, NMR, 14. Ketoglutarate, Structure, 15. Nematic, Nematic liquid crystal, 16. Actinobacillu, Serotype, 17. Bovine serum albumin, Human serum albumin, 18. Clostridium difficile, Bioterrorism, 19. Lactococcal, Lactococcus lactis subsp, 20. Virtual screening, Molecular, 21. Streptococcal, Erythrogenic, 22. Listeria monocytogene, Antimicrobial, 23. Electrospray, Matrix, 24. Biosynthetic, Biosurfactant, 25. Venom, Venomou,	

M8-Surgery	677,995
Patient, Gastric, Pancreatic, Pylorus, Case, Hepatocellular, Surgical, Colorectal	
0. Hepatocellular, Hepatocellular carcinoma, 1. Esophageal carcinoma, Esophageal, 2. Achalasium, Gastroesophageal reflux disease, 3. Pylorus, Helicobacter pylorus, 4. Rectum, Rectal, 5. Acute pancreatiti, Pancreatiti, 6. Portal, Portal hypertension, 7. Biliary, Acute cholecystiti, 8. Meckel, Diverticulum, 9. Pancreatic, Pancrea, 10. Colorectal, Colonoscopy, 11. Splenic, Peritoneoscopy, 12. Pyogenic, Intravascular papillary endothelial hyperplasium, 13. Gastric, Selective vagotomy, 14. Inguinal, Inguinal hernium, 15. Percutaneous endoscopic gastrostomy, Percutaneou, 16. Caustic, Biodegradable stent, 17. Gallbladder, Biliary, 18. Acute mesenteric, Superior mesenteric, 19. Foreign, Button, 20. Diverticular disease, Diverticular, 21. Liver transplantation, Biliary complication, 22. Sterilisatie, Retroperitoneal, 23. Biliary, Case report, 24. Parathyroid, Subtotal,	

P1-Applied Physics	4,103,886

Effect, High, Magnetic, Thin, Microstructure, Mechanical, Optical, Alloy
0. Alloy, Steel, 1. GaA, Molecular beam epitaxy, 2. Superconductor, Critical current, 3. Eu, Yb, 4. Microstructure, Mechanical property, 5. Ferroelectric, Dielectric property, 6. Diamond, Thin, 7. Silicon, Boundary, 8. Plasma, Laser, 9. MOSFET, High, 10. Graphene, Electronic, 11. Ceramic, Composite, 12. Magnetic, Magnetism, 13. Corrosion, Carbon steel, 14. Si, Surface, 15. Composite, Composite laminate, 16. GaN, SiC, 17. Solar, Amorphou, 18. Proces, Abrasive, 19. Topological, Quantum, 20. Gyroscope, Design, 21. Superalloy, Microstructure, 22. Amorphou, Metallic, 23. Resist, Plasma, 24. Magnetic, Magnetic property, 25. Solder, Sn, 26. Thermoelectric property, Thermoelectric, 27. Laser, Breakdown spectroscopy, 28. Molten, Steel,

P2-Astronomy	1,632,125
Cosmic, High, MeV, Nucleon, Cosmological, NGC, Finite, Gravitational	
0. MeV, Nuclear, 1. Circumstellar, Stellar, 2. Abelian, Nilpotent, 3. Cosmological, Cosmic, 4. Operator, Singular integral, 5. Brane, Supersymmetric, 6. Chiral, Decay, 7. Gravitational, Gamma ray burst, 8. NGC, Galaxy, 9. Nucleon, Higgs boson, 10. Algebra, Minimal, 11. Congruence, Prime, 12. Cosmic, Neutrino, 13. Ray astronomy, CCD, 14. Cyrillic, RHIC, 15. Kähler manifold, Metric, 16. Finite group, Finite, 17. Orthogonal polynomial, Asymptotic, 18. Series solution, Hyperbolic potential,	

P3-High Energy Physics	1,345,484
Optical, High, Photonic, Design, Low, Light, Electromagnetic, Fiber	
0. Antenna, Compact, 1. Photonic, Metamaterial, 2. Entanglement, Quantum, 3. Photonic, Optical, 4. Optical, Optic, 5. Low, Mixer, 6. Fiber laser, Fiber, 7. Electromagnetic, Design, 8. Optical, Coherent, 9. Bose Einstein condensate, Trapped, 10. Switched, Bit, 11. Hologram, Digital holographic, 12. Terahertz, THz, 13. Nonlinear, Nonlinear Schrödinger equation, 14. Semi infinite nonlinear crystal, Slow, 15. Trapped ion, Nm, 16. Photorefractive, Holographic, 17. Electromagnetic, Magnetic field, 18. GHz, Plasma relativistic, 19. Single, Diamond, 20. Light, Random medium, 21. Time reversal, Microwave, 22. Fluctuation, Chao,	

C1-Inorganic Chemistry	2,627,313
Effect, Electrochemical, Synthesi, Preparation, High, Poly, Photocatalytic, Catalytic	

0. Electrochemical, Preconcentration, 1. Poly, Epoxy, 2. Catalyst, Catalytic, 3. Zeolite, ZSM, 4. Photocatalytic, Solar cell, 5. Cellulose, Mechanical, 6. Lithium ion battery, Electrochemical, 7. Oxygen reduction reaction, Electrocatalytic, 8. ZnO thin film, Thin, 9. Optical, Optical property, 10. Gold nanoparticle, Silver nanoparticle, 11. Carbon nanotube, Nanotube, 12. Graphene, Graphene oxide, 13. Polypyrrole, Polyaniline, 14. Poly, Membrane, 15. Atmospheric, Luminous efficacy, 16. Superhydrophobic, Surface, 17. Solid oxide fuel cell, Solid, 18. Catalytic, Catalytic ozonation, 19. Ionic, Ionic liquid, 20. Electroless plating, Organic additive,

C2-Organic Chemistry	2,402,274
Synthesi, Ius, Reaction, Complex, Crystal, Crystal structure, New, Molecular	
0. Vibrational, Molecular, 1. Bi, CO, 2. Synthesi, Bicyclo, 3. Synthesi, Azido, 4. Organic, Organic thin, 5. Palladium, Synthesi, 6. Synthesi, Yl, 7. Ius, Copper, 8. Coordination polymer, Crystal structure, 9. Intense, Ion, 10. IIus, Bpy, 11. Crown, Arene, 12. Crystal, Yl, 13. Enantioselective, Asymmetric, 14. Fullerene, Endohedral, 15. Spectroscopy, Ne,	

E1-Biological Sciences	4,212,793
Effect, Soil, Sediment, New, New species, Coastal, China, Influence	
0. Tropical, Habitat, 1. Hydrological, Tropical, 2. Soil, Nitrogen, 3. Fish, Coral, 4. New species, Phylogenetic, 5. Arsenic, Soil, 6. Microbial, Nov, 7. Aerosol, Air pollution, 8. Adsorption, Aqueous solution, 9. Hymenoptera, Lepidoptera, 10. PAH, Soil, 11. Song, Male, 12. Anaerobic, Microbial, 13. Landsat, Remote, 14. Coastal, Sediment, 15. Groundwater, Water quality, 16. Biofuel, Combustion,	

E2-Civil Engineering	2,724,654
Numerical, Effect, Concrete, Experimental, High, Study, Flow, Analysi	
0. Turbulent, Turbulence, 1. Concrete, Seismic, 2. Biodiesel, Combustion, 3. Concrete, Reinforced concrete, 4. Solar, Thermal, 5. Numerical, Finite, 6. Heat transfer, Nanofluid, 7. Existence, Homogenization, 8. Beam, Vibration analysi, 9. Fractional differential, Nonlinear, 10. Fluidized, Granular, 11. Structural health monitoring, Structural, 12. Structural, Stochastic, 13. Gear, Planetary, 14. Numerical, Wave, 15. Gas liquid, Bubble, 16. Piezoelectric, Aerodynamic, 17. Lattice Boltzmann method, Drop, 18. Elastic, Boundary, 19. Numerical, Evaporative, 20. Deterministic, Turbulent flow, 21. Acoustic, Sound, 22. Coal, Low rank coal, 23. Asphalt, Asphalt mixture, 24. Centrifugal, Centrifugal pump, 25. Flow, Cylinder, 26. Variational, Banach space,	

E3-Geology	1,777,075
Seismic, Tectonic, Volcanic, Southern, Geological, Application, Effect, Crustal	
0. Reservoir, Hydraulic, 1. Archaeological, Forensic, 2. Geochronology, Tectonic, 3. Seismic, Pile, 4. Ordovician, Stratigraphic, 5. Earthquake, Seismic, 6. Roman, Late antiquity, 7. Last, Climatic, 8. Seismic, Reverse time migration, 9. Sandstone, Rock, 10. Mar, Moon, 11. Crustal, Seismic, 12. Landslide, Debris flow, 13. Plasma mass spectrometry, Kunlun, 14. Volcanic, Volcano, 15. Hydrothermal, Albite, 16. Shale, Chlorophyll, 17. Earth, UNCLO, 18. Gas hydrate, Seismic,	

E4-Agriculture	981,653
Effect, L., Plant, Genetic, Growth, Maize, Rice, Identification	
0. Genetic, BB, 1. Drought, Abscisic, 2. Trichoderma, Phytophthora infestan, 3. Photosynthetic, PCC, 4. Fireblight, Plant, 5. Plant regeneration, Vitro propagation, 6. Cytokinin, Gibberellin, 7. Cell suspension culture, Flavonol, 8. Viru, Viroid, 9. Olive, Apple, 10. Aflatoxin, Deoxynivalenol, 11. Cadmium, Iron, 12. Arabidopsi, Xyloglucan, 13. Nitrate, Nitrate reductase, 14. Rhizoplane, Metarhizium, 15. Grain yield, Sunflower, 16. Bacillus thuringiensi, Transgenic, 17. Starch, Nonglutinou, 18. Camelina, Fatty acid desaturase gene,	

E5-Energy	817,659
High, Power system, Current, Analysi, Design, Electric, Converter, Inverter	
0. Converter, DC DC converter, 1. Permanent, Reluctance motor, 2. Power system, Available transfer capability, 3. Transformer, Partial, 4. Smart, Electric vehicle, 5. Solar, Photovoltaic system, 6. Induction motor drife, Brushless DC motor, 7. Harmonic, Current harmonic, 8. Distributed generation, Distribution network, 9. Transformer, Considerable, 10. Multilevel inverter, Inverter, 11. Optimal power flow, Hydrothermal, 12. Hybrid electric, Electric, 13. Induction generator, Ampere, 14. Inverter, Islanded, 15. Insulator, Composite insulator, 16. Photovoltaic, Partial shading condition, 17. MMC, Modular multilevel converter, 18. Wireles, Wireless power transfer, 19. Thermal, Modelling, 20. Railgun, Metal, 21. Induction machine, Wound rotor induction machine,	

Table T2. The list of excluded data The domains-year-nation with dynamically changing research topics were excluded from the analysis. The TPI assumes that the foremost research topic is constantly changing in each nation. For this reason, TPI cannot be applied to domains where the research topic is dynamically changing. We excluded the data for the domains-year-nation for which the research topics changed dynamically by applying the following two criteria: 1) Mean distance to others (the ratio of average cosine similarity to other nations' $\mathbf{T}$ in a year to that of other nations' $\mathbf{T}$ in the same year) is less than 80% of that of the previous year. 2) The nation's mean distance to others in the year is greater than 0.02. This condition excludes nations whose topics differ significantly from those of other nations. Based on these conditions, the following dynamically changing domains-year-nation were excluded.

The research topics of nine nations in the cluster M4-Infectious Diseases in 2020 changed dynamically. COVID-19 had a significant impact on this domain. We excluded all data for seven infectious diseases in 2020 from our domain-specific analysis. In other combinations, data for a single nation change dynamically over the course of a year. For other domains-year-nation combinations, we excluded the detected nation's data for the changing domain within the given year (not all domains for the nation for the given year).

Domain	Year	Nation
CS1-Computer Science	2003	France
M4-Infectious Diseases	2020	China
M4-Infectious Diseases	2020	France
M4-Infectious Diseases	2006	Hong Kong
M4-Infectious Diseases	2020	India
M4-Infectious Diseases	2020	Iran
M4-Infectious Diseases	2020	Italy
M4-Infectious Diseases	2020	Spain
M4-Infectious Diseases	2020	UK
M4-Infectious Diseases	2020	US
M6-Orthopedics/Dentistry	2020	India
P2-Astronomy	1992	Switzerland
P3-High-Energy Physics	2003	France
P3-High-Energy Physics	2009	France
E5-Energy	2007	Canada
E5-Energy	2003	France
E5-Energy	2009	France
E5-Energy	2014	India
E5-Energy	2019	India
M8-Surgery	2000	Netherlands
